# Exploring the Dynamics of Canine-Assisted Interactions: A Wearable Approach to Understanding Interspecies Well-Being

**DOI:** 10.3390/ani14243628

**Published:** 2024-12-16

**Authors:** Timothy R. N. Holder, Colt Nichols, Emily Summers, David L. Roberts, Alper Bozkurt

**Affiliations:** 1 Department of Aeronautics and Astronautics, Massachusetts Institute of Technology, Cambridge, MA 02139, USA; tholder@mit.edu; 2 Department of Electrical and Computer Engineering, North Carolina State University, Raleigh, NC 27695, USA; cwnicho2@ncsu.edu (C.N.); emsummer@ncsu.edu (E.S.); 3 Department of Computer Science, North Carolina State University, Raleigh, NC 27695, USA; dlrober4@ncsu.edu

**Keywords:** canine-assisted interactions, wearable systems, psychophysiology sensors

## Abstract

Canine-assisted interactions (CAIs) have been explored to offer therapeutic benefits to human participants in various contexts, from addressing cancer-related fatigue to treating post-traumatic stress disorder. Despite their widespread adoption, there are still unresolved questions regarding the outcomes for both humans and animals involved in these interactions. Previous attempts to address these questions have suffered from core methodological weaknesses, especially due to absence of tools for an efficient objective evaluation and lack of focus on the canine perspective. In this article, we present a first-of-its-kind system and study to collect simultaneous and continuous physiological data from both of the CAI interactants. Motivated by our extensive field reviews and stakeholder feedback, this comprehensive wearable system is composed of custom-designed and commercially available sensor devices. We performed a repeated-measures pilot study, to combine data collected via this system with a novel dyadic behavioral coding method and short- and long-term surveys. We evaluated these multimodal data streams independently, and we further correlated the psychological, physiological, and behavioral metrics to better elucidate the outcomes and dynamics of CAIs. Confirming previous field results, human electrodermal activity is the measure most strongly distinguished between the dyads’ non-interaction and interaction periods. Valence, arousal, and the positive affect of the human participant significantly increased during interaction with the canine participant. Also, we observed in our pilot study that (a) the canine heart rate was more dynamic than the human’s during interactions, (b) the surveys proved to be the best indicator of the subjects’ affective state, and (c) the behavior coding approaches best tracked the bond quality between the interacting dyads. Notably, we found that most of the interaction sessions were characterized by extended neutral periods with some positive and negative peaks, where the bonded pairs might display decreased behavioral synchrony. We also present three new representations of the internal and overall dynamics of CAIs for adoption by the broader field. Lastly, this paper discusses ongoing options for further dyadic analysis, interspecies emotion prediction, integration of contextually relevant environmental data, and standardization of human–animal interaction equipment and analytical approaches. Altogether, this work takes a significant step forward on a promising path to our better understanding of how CAIs improve well-being and how interspecies psychophysiological states can be appropriately measured.

## Simple Summary

This study utilizes electronic sensors to investigate the outcomes of Canine Assisted Interactions (CAI), a growing therapeutic field, for both human and animal participants. It represents the first attempt to deploy synchronized wearable systems on both humans and dogs, allowing for the continuous and simultaneous collection of physiological and behavioral data during interactions. Leveraging this data, the research examines the real-time dynamics of CAIs, moving beyond traditional survey-based pre- and post-session evaluations. Three innovative visualization tools—a subsession heatmap, a synchrony table, and a metric correlation matrix—are introduced to better characterize the interactions and bonding within human-dog dyads. Preliminary exploratory analyses provide insights that inspire further investigation into CAI mechanisms. This research marks a significant step forward in using multimodal data collection to deepen our understanding of human-animal interactions, particularly in therapeutic settings.

## 1. Introduction

Canine-assisted interactions (CAIs) are a class of widely adopted complementary and alternative medicines that utilize interactions with trained dogs. Like all animal-assisted interactions (AAIs), CAIs aim to improve quality of life, which is reflected in specific clinical endpoints (e.g., blood pressure, cortisol, etc.), for human participants [1]. There has been a wide variety of outcomes, from no effects or neutral effects to certain benefits indicated for human participants [2]. Many of these positive effects are attributable to bonding between the interactants or to second-order effects of the interactions (e.g., exercise, external focus, etc.), among other things [2]. In trying to better understand the nature and source of the observed benefits, CAI researchers have recently been moving towards objective and quantitative evaluative methods and away from more qualitative, subjective approaches. Wearable sensor systems for dogs open up opportunities to glean greater understanding of the effects of human–canine interaction [3,4]. However, both the measurement tools for and the targets of this quantification would benefit from additional research and development.

While the use case of dogs interacting with humans in controlled environments is the most common pet therapy, many studies focus only on quantifying the human element, generally neglecting the dog’s perspective and limiting the depth of interspecies interaction investigation possible [5,6,7,8]. This not only has ethical implications—in the event that the selected therapy negatively impacts the dog participant—but also affects the quality of the therapy, which is highly dependent on the well-being of the therapy animal [2,9,10]. Similarly, CAIs tend to focus on general assessments of quality of life, but the high variability in the measures used and the outcomes observed in these assessments can partially be attributed to the vagueness of the typical quality of life concept. To address these first two concerns, we propose switching to a dyadic psychophysiological perspective. Psychophysiology generally refers to the idea that mental and emotional processes have detectable physiological correlates, and it provides a more solid theoretical framework for objective interpretation of quantitative CAI data [11,12,13,14]. Additionally, by focusing on both members of the interacting dyad, this perspective allows for direct or comparative measurements that the general quality of life approach does not focus on, such as those from human or animal subjects with limited communication or non-existent survey-response capabilities [15]. The existing studies specifically used activity levels to track sleep quality in interspecies bedfellows only.

When considering quantitative data collection, some CAI researchers have incorporated biochemical assays (e.g., measuring oxytocin, vasopressin, or cortisol) and electronic monitoring devices (e.g., measuring heart rate or blood pressure) into their studies, representing a major step in the right direction. As CAIs can range in duration from 10 min to 16 h and in activity from quiet stroking to vigorous physical movement, many of the current tools confine measurement to pre-intervention and post-intervention data collection and further limit these collections to clinical or research settings [2]. Common psychophysiological measurements using the available biochemical and electronic monitoring technologies can be very physically invasive and also tend to significantly impact or obstruct CAI activities. To address these concerns, we deployed a study design that eliminates the need for biochemical analyte collections. We utilized a wireless wearable electronic monitoring system developed by our research group—hereafter referred to as SySy (synchronized system)—for the continuous and non-invasive measurement of human and canine physiology simultaneously, as described in Part I: Sensor System Development [16,17,18,19,20]. In Part II: Pilot Study and Proof of Concept, we present an initial experiment using this wearable SySy for quantitative psychophysiological analysis of an interspecies CAI dyad. Though nascent, this work contributes to field efforts aiming to better include and quantify the canine perspective in CAI research and to improve animal-centered emotion-recognition technologies for real-world deployment [21,22,23]. The tools, methods, and results in this paper may eventually enable researchers to better and more consistently connect CAI inputs to outcomes, to identify relevant psychophysiological states in dyad members, to conduct studies validating CAIs as viable complementary therapies, and to increase the benefits of CAIs for humans and animals alike as they interact in various contexts. The products of this work may also significantly bolster studies in other human–animal interaction scenarios by laying the foundational principles for real-life human and animal data collection beyond the research environment. Altogether, this article presents a unique study integrating and deploying multiple wearable systems on both interactants in a typical CAI while synchronously collecting multimodal psychophysiological data and analyzing it in comparison to simultaneous survey and behavior coding ground truths.

**Part I: Sensor System Development** 

## 2. Overview of the State of the Art and Design Process

In order to fully motivate the need for an integrated approach to quantifying interspecies interactions and to introduce an ergonomic, validated synchronized system for addressing this need, we first reviewed the CAI field’s research and the broader human–animal interaction (HAI) space’s commercially available devices for human or canine psychophysiological measurement. The main questions of this mini-review were what objective, quantitative, non-invasive (OQN) methods (largely electrophysiological measures) are used in AAI research and to what in the non-OQN space they are experimentally correlated (e.g., biochemical analytes, psychological surveys, behavioral coding, etc.).

There were five main results from this investigation of the literature and beyond [2]. Firstly, of the many tools and methods to evaluate CAIs and HAIs, the most-often-used are behavioral coding, biochemical assays, and psychological surveys, though non-chemical physiological means, such as heart rate and blood pressure, are also becoming more represented. Secondly, there are several target substrates for evaluation in HAIs broadly and AAIs specifically, with the most common by far being electrocardiography (ECG) (especially heart rate/variability derivatives), oxygen saturation, respiratory activity, and blood pressure. Thirdly, in keeping with major aspects of the “framework for selecting and benchmarking mobile devices in psychophysiological research”, as described in [24], we found the space to have 222 CAI-applicable devices to detect the most common physiological targets, though “canine only” systems were severely under-represented and only 37% were supported by a complementary/validating scientific publication. Furthermore, of the system prices in USD as of 2023 that we could accurately determine by surveying 129 different devices, the mean cost was close to 150 USD in a range of 5–1700 USD [25]. Noting that these commercially available systems measure only one or two signals each, this reflects the relative expense and limitations of using commercial devices, either individually or in combination with others, in CAI contexts. Fourthly, several objective, quantitative, and non-invasive (OQN) parameters show significant change independently and/or good correlation with validated behavioral or psychological measures. For example, heart rate often significantly decreases, systolic blood pressure often significantly decreases, and diastolic blood pressure sometimes significantly decreases with human–animal interaction. Additionally, positive or negative movements in these two cardiovascular metrics tend to be reflected in accompanying survey instruments, but none, including other measures beyond heart rate and blood pressure, have been consistently correlated across studies [1,26]. Lastly, it is noticeable that the tools utilized throughout HAIs are generally non-specialized for their respective use cases. Many tools are adapted from other targeted human application spaces and often ignore ergonomic, experimental, and analytical peculiarities unique to human–animal interaction scenarios. On a major related note, the animal’s perspective is often not considered or centered in the design/development of OQN devices. For these non-human participants, the main measures are heart rate or movement, if collected at all. Furthermore, having environmental- and interaction-specific sensors (e.g., barometric pressure, intersubject proximity) may provide additional context for large-scale physiological, behavioral, and psychological survey data sets that appropriate data science methods may be able to detect and isolate. In other words, the full picture includes the human, the animal, their interaction, and the environmental context.

These reviews also reiterated that in HAI there is much variation in study design, which is accompanied by lack of controls and general measurement method inconsistency. While targeted outcomes and study approaches may vary reasonably, the field could benefit greatly from a core system and modular protocol developed with HAIs specifically in mind. It is clear that the issues in study design and endpoint measurement stem from methodological limitations and the feasibility of various tools for use with animals. This brief overview provides researchers with some understanding of the tools that are currently commercially available, and it allows them to identify some promising tools that could be added to future OQN system development efforts. To further advance the field, a more granular look into the interaction is required, along with the objective evidence base to gain acceptance in medical communities, by insurance providers, etc., and the versatility to evaluate a broader spectrum of subject outcomes. Methods that are objective, quantitative, and non-invasive are the minimum needed to advance these objectives and move the AAI field beyond the very common general tests of feasibility, comparisons between treatment groups, or broad attributions of positive outcome from pet interactions.

### 2.1. Stakeholder Survey

The surveying of the literature provides a solid idea of the general problems in the AAI, CAI, and HAI spaces, mostly centered around the standardization of experimental protocols and the efficiency of measurement tools, as well as some of the components necessary to address them, such as dyadic, multimodal research, and commercial systems. While there are thousands of published AAI studies, most pet therapy programs occur without a specific publication record. Therefore, we conducted a brief survey study that solicited responses and opinions from CAI stakeholders (i.e., researchers, facilitators, participants, patient family members, therapy dog handlers, and any other individual involved in CAIs). This was done with a combination of an 8-point Likert scale and 14 short-answer questions asked of 10 respondents via four sections of an online survey approved by the NC State Institutional Review Board [see Table S1 in Supplemental Materials]. With Likert responses of 1 and 5 indicating the negative and positive extremes, respectively, ratings of 2 or 4 conveying the sentiments of ‘somewhat negative’ or ‘somewhat positive’, and 3 representing a neutral response, only the response means for six questions out of eight were above neutral. The exceptions were the questions about satisfaction with the current methods and with the outcomes of CAIs for dogs. This echoed the review results in previous sections, where researchers were able to proceed with their work somewhat, but consistently called for methodological improvements and more canine emphasis. Also worthy of note are the responses to the two last questions, which indicate that the respondents more than ‘somewhat’ believed that environment greatly affects CAIs, and that they were optimistic about the future of CAIs. This is an encouragement for device and system developers to address the gaps in the field and also to incorporate ambient, environmental, and other contextual signals into their development processes.

The remaining non-Likert sections of the stakeholder survey asked questions about how to best identify psychophysiological states in CAIs, about the context and design of CAIs, and about the respondent’s thoughts on our custom SySy design plans after a contextualization primer [see Table S2 and Figure S1 in Supplementary Materials Section S1]. Responses to open-answer questions were evaluated thematically, and the most salient trends are reported [27,28].

Question 9 asked about the indicators of psychophysiological states in dogs. In keeping with the literature [29], the respondents generally identified a relaxed body, tail wagging, and engaging approach behaviors as primary positive indicators, and a tense body, avoidant behaviors, tail tucking, yawning, lip licking, and “whale eyes” as key negative indicators. Some of these indicators, like tail wagging, are better-suited for assessment by OQN measurement systems, while others, like “whale eyes”, may be more complicated to consistently detect. When asked in Question 10 about past experiences measuring psychophysiological states, the responses were grouped around the themes of using observation over extended time deltas, behavioral coding, or checklists, and some use of ECG, salivary cortisol, or the owner report. Confirming the general tenor of the literature, half of the respondents cited little-to-no experience here. The Question 11 responses strongly centered on non-invasive wearables for dogs and video assessment tools as the most desired additional needs for use in CAIs. The respondents also indicated interest in validated written assessment tools, protocols for individualized psychophysiological state assessment, and more handler training in assessing their therapy dog’s state. The Question 12 responses about desired data parameters centered on exportable, objective data in an intuitive format for non-technical users. One respondent, identifying herself as a program director, stressed the “objective” component as she currently relied on participant anecdotes and staff opinions to evaluate CAIs, and she noted that she could greatly benefit from standardized methodologies. Otherwise, the respondents were largely interested in making decisions about the success of the dog in the CAI context (e.g., duration and intensity of interactions, suitability for the task, match to patient, etc.)

Questions about the designs of CAI experiments and the contexts in which they occur revealed three key themes. Firstly, the dogs, handlers/owners, and clinical staff were most consistently present during the interactions, which lasted between 30 and 60 min and could occur in contexts ranging from schools and clinics to busy hallways and lounge spaces. As most CAIs are modeled as an interaction between a human and a dog, psychophysiological theories should be updated to include between one and two individuals beyond the human subject and the therapy dog (i.e., the dog handler and, potentially, a researcher/clinician). Next, the respondents expressed interest in the integration of measurement systems into existing dog attire (e.g., collars, vests, etc.) and for human systems to be non-distracting and to maintain privacy. Lastly, the CAI activities most likely to induce positive psychophysiological states were, for the therapy dog, (a) gentle petting, (b) exercise and play, (c) feeding and treats, (d) calm vocalizations, and (e) copious breaks, and, for the human interactant, (1) touching the dog, (2) having a good conversation with the handler, (3) seeing a happy dog. These items could all be considered for incorporation into a modular CAI protocol aimed at the maximum benefit for the human and animal interactants. These responses also reaffirm the notion that the dog’s experience affects the quality of the interaction for the human.

The last survey question asked for general recommendations, and, as such, the responses varied. Two experimental suggestions included (a) testing our custom SySy in individual interaction settings vs group CAIs, and (b) recruiting diverse human subjects (as certain populations are very unrepresented in CAIs research) as well as diverse canine subjects (citing a relative over-representation of golden retrievers). One respondent expressed dissatisfaction with an existing commercial pet wearable while another implored us to focus heavily on the canine perspective since humans can speak for themselves. This interrogation of stakeholder’s opinions was very informative for the development of both a modular protocol as well as objective, quantitative, and non-invasive systems for CAI and psychophysiological state assessment. Overall, the respondents mirrored many of the thematic issues highlighted in the literature reviews, and they were, generally, sanguine about the future of our lab’s proposed SySy and of the CAI field, in general.

### 2.2. Device/System Characterization

Due to the absence of a single commercially available system that could gather all the relevant data streams simultaneously and synchronously, we assembled a custom system composed of commercially available integrated circuits and sensors to work in concert to quantify CAIs. The core advantages to this approach are access to most data without filtering through proprietary algorithms, higher sampling rates, power optimization, increased intuitiveness for non-technical users, and a modular, wearable test bed that is functional even if or as individual devices are added or removed. All these make up a custom application-specific system to better suit given AAI scenarios.

**Figure 1:**
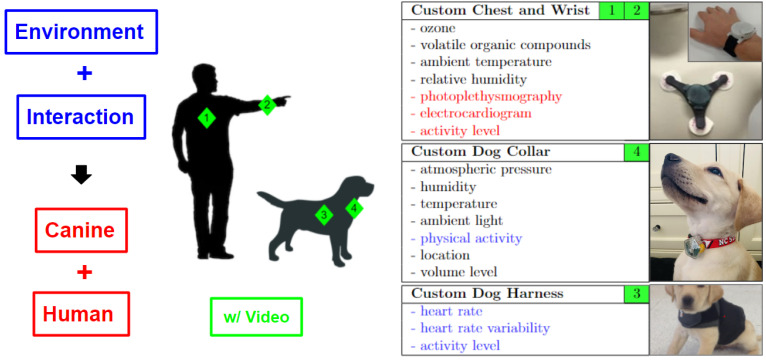
Custom-designed wearable research system devices, representation, and data streams. The chart on the left depicts the involved CAI data streams. The dog and the human silhouettes depict the location of each system device. The representative pictures and paired lists further depict each system’s location and sensors.

The custom-assembled sensor system (Figure 1) includes a suite of two wrist-worn sensors and one chest-attached sensor for the human participant. One of the wrist-worn devices is a commercial sensor for collecting electrodermal activity and skin temperature (Empatica E4). The other wrist-worn device was developed at NC State and includes photoplethysmography (PPG) and inertial measurement sensors in the form of an accelerometer to characterize wrist movement. The next generation of this system will include skin temperature and electrodermal sensors to eliminate Empatica E4 completely, using a single-wrist system. The chest strap includes an ECG-based heart rate monitor and another accelerometer for the chest movement [18]. For the canine participant, the system assembled includes a harness for torso movement and an ECG sensor for heart rate tracking [30]. This is coupled with a collar device for another accelerometer on the neck region of the dog [31]. Each device in the system is supported by non-adhesive, custom-designed electrodes and communicates with a data aggregator via Bluetooth [25]. The custom-designed human and canine sensors also include environmental sensors that were not used for this study, as is explained later. These include ozone, volatile organic compounds, relative humidity, barometric pressure, ambient temperature, noise, and light. All these five systems (three for humans, two for dogs) work with an iOS smartphone app to stream data continuously and synchronously before storage to the cloud [32,33,34]. The canine harness includes a neck strap stabilizer with pockets and through-holes for wiring and electrode-to-skin access without shaving fur [17].

It is important to note that all of the discussed research systems are robustly designed, as recommended by experts, stakeholders, and the literature, for use in movement contexts and/or to be deployed in canine-involved scenarios [35]. Once assembled, bench testing and characterization of the devices of the combined wearable system was necessary, to ensure its operationality and suitability for CAI purposes. For this effort, we used the “Standard In-lab Testing” (SIT) protocol previously developed by our technical team, wherein (1) each sensor on each device was evaluated, (2) each device’s connectivity was interrogated, and (3) each device’s battery charge and discharge characteristics were noted [36].

For heart rate (HR) validation, we used a bulky and large commercial device (Go Direct™ EKG Sensor, Vernier Software & Technology, Beaverton, OR, USA) as ground truth. The heart rate, determined from the various ECG sensors, was assessed during a 4 min human and canine subject deployment [Table S3] and benchmarked against the ground truth system, with an absolute error of 0.36% and 0.64% for humans and dogs, respectively. The estimates and interpolation of HR from PPG, with respect to the Vernier, caused an absolute error of 6.19%. The gravitational pull and rotation of devices along three orthogonal axes during characteristic movements provided the necessary controlled acceleration for activity-level assessment. The general connectivity prominence behavior of each device was tested by measuring the received signal strength indicator (RSSI) of the BLE signal at various distances from the connected iOS or computer device, using the nRF Connect Analyzer iPad app. We also tested the wireless connection range of each device in one or more environments (i.e., in an open field outside, in a typical house with many rooms, and in a long office building hallway). Finally, we also assessed the median device battery life and charging duration through timed charging–discharging cycles. Individual sensor and connectivity range test representative examples can be found in [36]. Tables S4–S6 summarize the remaining devices’ connectivity, signal strength, and battery life results, respectively. Since typical CAIs range from 10 to 60 min with a median value of 20 min and occur within approximately 20 ft-by-20 ft spaces, our integrated wearable system is sufficient to provide high-quality quantification of the relevant signals in these interactions [33,36,37,38,39,40].

### 2.3. Ergonomics

The next stage was to comparatively verify the ergonomic viability of the assembled wearable systems for both the human and canine participants, in context. Developing a protocol to measure the animal wearability was boiled down to an ethnographic and literature survey. Notably, a scan of various literature databases found very few papers that focus on canine ergonomics—including that of working military, police, or service dogs. It revealed an insufficient treatment of the subject, and it represents a major gap in this research space [41]. An important exception is the work presented in [42], which thoroughly discusses animal ergonomics. The study presented in [43] argues the inability of humans to avoid anthropocentrism and the consequent exploitation of animals as the core reason, where humans create a device without animals directly being involved in the process. Another study [44] discusses the guiding principles for animal-centered systems, after observing the reactions to certain devices. Aspects like sensory imperceptibility, physical unobtrusiveness, and cognitive unacceptability are all important factors in the wearability of the devices for non-humans. Differences in the behavior of an animal before and after wearing the device illustrate the animal’s judgment of the device. This approach—confirmed by our stakeholder survey experts, as well—was the primary evaluative paradigm we employed for dogs. By working within an animal-centered framework, we hoped to minimize the human-centered pitfalls that are inevitably intertwined with animal technology.

On the human side, it was decided that a mixture of rapid upper limb assessment (RULA), rapid entire body assessment (REBA), and ethnographic research would provide the best ergonomic measures for humans, though we also considered the Borg, comfort rating, psychophysiological neuroergonomics, and many other wearability scales [45,46,47,48]. Our approach was to first evaluate how people had evaluated human wearable devices across contexts (e.g., occupational, industrial, etc.) and to review maps of possible device placements/configurations on human bodies [49]. From the aforementioned resources and searches, we extracted the relevant considerations for humans (and, as appropriate, canines), focusing primarily on wearability, usability, and safety. As an example, these considerations for canines included device weight-to-body mass ratio, safety, general size/bulkiness, surface area covered (noted in the stakeholder survey), and easy integration into existing attire. For humans, the foci were comfort, required additional energy expenditure, level of anxiety induction, propensity for physical or mental distraction, feasibility of usage in combination with other devices, etc., [50]. Developing survey questions that incorporated these general and specific considerations culminated in our ergonomics test protocols for humans and for canines, with the accompanying survey instruments. Each survey was expected to be taken by the wearer after a period of directed exercise and movements or after use in an experimental session, and they are herein reported as aggregated Likert scale responses or thematically summarized open-ended feedback [see Figures S3–S5]. We also compared our custom SySy prototype with an assembly of commercial off-the-shelf wearables for humans (including Qardio arm, MUSE headband, Biostrap, GoBe2, Polar H10, Zephyr Belt, Oura Ring) and dogs (MeasureON!).

To evaluate the ergonomics for wearable devices, we collected several ratings of the various human and canine wearable device candidates from two human subjects, using the test protocol and ergonomics surveys [Table 1 and Table 2]. These demos focused on how the system worked in an animal-involved scenario (e.g., ease of setup, wires affecting interaction, animal toleration, data export). The human wearable devices tested scored in the C and B range with the exception of the Empatica E4 [Table 1]. The main fault of the rated sub-A systems tended to be in the General Questions section asking about comfort during wear, ease of set up, and not generating negative emotions. Non-Likert scale comments spoke to many devices’ difficulty in exporting raw or any data, provided descriptions of exactly how movement was limited, and often re-emphasized discomfort. For Empatica E4, the only major comments concerned its general bulkiness, relative to the wrist, and the fact that it must be worn sufficiently tightly to maintain contact with the electrodermal activity and skin temperature electrodes. The only commercially available canine wearable device physically tested was the VetMeasure MeasureON! System that earned a D rating ergonomically but brought useful additional measures to the table (i.e., canine skin temperature) [Table 2]. The comments here centered on the system’s difficulty to set up and the discomfort to some dogs caused by the comb-like metal electrode prongs. Generally, the commercially available wearable devices evaluated have limited adjustability and, thus, must be pre-selected or subsequently modified to best fit the range of human and canine sizes.

Overall, evaluating the wearable devices ergonomically was very informative, with some notes. Many devices had very low sampling rates (i.e., one measure every 2 to 5 min), difficult setup/affixing protocols, and non-transparent data-processing pipelines. These opaque processes involved proprietary algorithmic manipulations of the data that we could not interrogate as to their validity, efficiency, or effectiveness for our purposes. That said, some commercially available systems have the added benefits of being widely cited in the scientific literature and/or having officially associated analytical pipelines with smooth data processing (i.e., Empatica E4, MetaMotionR).

For the individual and combined ergonomics of our SySy research prototype, we also asked the human participants (from the experiment detailed in Part II: Pilot Study and Proof of Concept) to complete the full human and canine ergonomics evaluation forms after every full session. From these evaluations, the custom wrist band and chest patch research systems scored in the A and B range, respectively. The comments about these systems centered around dealing with gel-based ECG electrodes and other medical tapes for affixing the chest patch enclosure, device stiffness, and some limitation of wrist mobility for the wrist band [Table 1]. The custom canine harness and smart collar research prototype individually scored in the C and A+ ranges, respectively. The latter score was due to the collar unobtrusively clipping onto the dog’s collar—a very seamless integration into existing attire—with only one comment noting some very minimal limitation in brushing the dog’s neck area [Table 2]. The harness rater subtractions and comments occurred due to the Velcro interacting with fur, especially when the dog rubbed against non-living objects (like carpets or clothing), canine discomfort with ECG electrode gels, and limitation of some of the surface area for petting when very small dogs wore the custom harness system.

In combination, the SySy faired well from a human perspective, as our initial attempt at an integrated synchronized system for quantifying interspecies interactions. It scored an A with comment themes reiterating the aforementioned difficulties with adhesives and wrist motion. Additional notes were that for dogs that like to jump up on the chest there is some unavoidable chance that human torso-borne devices can become dislodged and that, for small dogs, the interaction of wrist devices with canine harnesses can be somewhat limiting. From the canine perspective, the combined SySy collar and harness also scored well (i.e., A-), with similar comments focused on exposed Velcro catching other fabrics, on the inability of dogs to reach itches under the harness, and on the general limitation of petting real estate. Throughout these pilot test interactions, we also tested the ergonomics and data collection of a diaper-borne intertial sensor to detect tail wagging [Figure S6] [51]. For this, some dog owners noted that their dogs did not like the diaper and that this approach should be modified/resized somewhat to allow for more fur and canine private area freedom.

In our design process, two changes were implemented on the SySy prototype, based on these ergonomic evaluations. Firstly, we started to use more user-friendly tapes and straps for attaching the custom chest patch to the human body, in order to better accommodate female subjects and to increase comfort. Secondly, as the majority (71.9%) of canine subjects had long or medium-length fur, which can interact poorly with Velcro, we designed an alternate harness option for housing the electronics, which was made of stretchable, rayon fabric [see Figures S7 and S8 in Supplementary Materials Section S3]. This change kept the adjustability of the main custom harness prototype, reduced the bulkiness and surface area covered, and eliminated the use of Velcro (by tying off the ends of the fabric).

**Part II: Pilot Study and Proof of Concept**

In order to advance the state of the practice in CAI measurement, the previous section, titled as “Part I: Sensor System Development", briefly described our efforts to review scientific and commercially available wearable sensor systems, collect CAI stakeholder needs, characterize the included devices, and evaluate the systems’ ergonomic impact in context. Together, these efforts resulted in an iterated SySy design and prototyping process for CAIs. This system was used throughout the pilot test described here in Part II.

### 2.4. Pilot Study Objective and Theoretical Background

In this section, we present a pilot study to deploy our SySy prototype and to analytically probe the relatively higher-resolution dyadic data collected, from a CAI context. We were interested in the investigation of the following questions: (i) Does the interaction lead to an increase in positive psychophysiological states across all relevant measures when compared to a neutral, non-interaction period for the human and for the canine? (ii) Does the human–animal bond measurably exist, does it grow stronger with time together, and can it be peripherally detected with the wearable system’s physiological data? (iii) Do human–dog interaction inputs (e.g., human touches dog; dog licks human) lead directly to the observed AAI outcomes (e.g., from surveys; in clinical endpoints), and are the data collected continuously capable of demonstrating these connections?. As with any pilot test, the evaluation of these questions was preliminary and highly contingent on experimental execution and data quality.

Our approach was motivated by the core psychophysiological framework, which suggests that mental and emotional states (such as stress, bonding, and flow) have physiological correlates in animals, which are context- and stimulant-dependent [19,20,52]. Affective states are multifaceted events that recruit multiple bodily systems, from the neural to the endocrine systems, and they are best approximated by fusing and correlating multimodal data streams from several related sources [20,29,37,52]. For the purposes of this pilot test, a positive psychophysiological state was defined as a composite of the positive dimensions of each of the included modalities (i.e., survey, behavior coding, physiological data), as determined by the interrogations of the literature. As posited by several CAI mechanistic hypotheses, we assert that positive human–animal interactions can lead to dyadic relationships that can then encourage human–animal bonds via mutually beneficial quality time and positive contact [53,54]. Finally, we see potential in behavioral and physiological synchrony as a burgeoning metric of bonding between species [55,56], and we also see potential in heart rate, heart rate variability, and physical activation as relevant indices of human and canine well-being [29,55,57,58].

Overall, we aimed to use the same devices and systems across a number of individuals, to collect data that would provide a unique perspective when compared and correlated to each other. This would produce generalizable results and could help persuade the broader CAI field to use standardized protocols, systems, and analytical methods to increase the comparability of data across studies [59]. Specifically, this approach advocates the adoption of the integrated tools developed and described herein—and, potentially, the non-contact or camera-based ones described elsewhere [60,61]—by other AAI studies outside of the laboratory and/or for use by agricultural or domestic animal-involved stakeholders. An additional goal of this pilot study was to differentiate between positive, negative, and neutral psychophysiological states in the context of brief CAIs, initiating and lubricating the transition from anecdote or quality of life measures to more objective approaches [62]. This would then enable large randomized controlled trials that would further breakdown the macro-psychophysiological state categories of positive, neutral, or negative into distinct categories (like fear, joy, etc.) and/or spectra (like valence, arousal, etc.), as desired by researchers [62,63].

## 3. Methods

### 3.1. Study Procedure

This study was granted the following ethical aprovals: NC State University Institutional Review Board (IRB) protocol numbers 20810 and 24393, NC State University Institutional Animal Care and Use Committee (IACUC) protocol numbers 24135. Our pilot study included a convenience sample of 8 adolescent/young adult humans (female = 62.5%) and 4 canines (female = 25%; breeds = Shih-Tzu and Maltese mix, Pitbull and Lab mix, Pitbull, and Yorkshire Terrier). The subject inclusion/exclusion criteria were as follows: (A) at least one of the human participants must have owned the participating dog for 6 or more months; (B) the dog must tolerate both collars and harnesses well; (C) the human must be willing and able to wear devices on both wrists and on the chest for roughly an hour [64]; (D) the human subject must be able and willing to complete both written/online surveys; and (E) both members of the dyad must be able and willing to come to a dedicated NC State University research lab space for data collection on at least two different days. Altogether, the recruited human and canine subjects, variously paired, completed 22 experimental day sessions.

**Figure 2:**
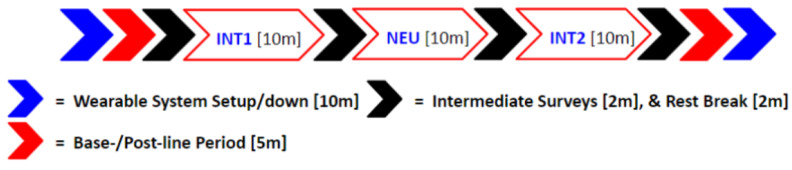
Pilot test protocol flow chart [72]. INT1 = interaction session 1; INT2 = interaction session 2; NEU = neutral session.

The pilot test was performed using an exercise research facility at NC State University. The room was carpeted, climate controlled, and isolated from noise or interruption by encompassing walls, a door, and floor-to-ceiling shades over the windowed areas. The human and dog participants were allowed time to become acquainted with the environment while the researcher went through the process of initiating the wearing system for data collection. The interactions were unstructured and largely human-seated/non-ambulatory interactions with the dog (e.g., talking to, touching, grooming, toy play, treat giving, commands, etc.) without the researcher in the room. It was left to the subject to determine whether to keep the dog leashed during the interaction, and most, but not all, opted for this setup [59]. As described below, survey instruments were administered before and throughout the experimental subsessions. The researcher would keep time and enter the experiment room to communicate the transition to a new subsession but would otherwise leave the participants unmonitored and uninterrupted. The 10 min interaction sessions were couched, before and after, in neutral sessions for the humans to sit quietly, alone, and relax (e.g., by reading a book, meditating, listening to music), with the dog being removed from the research space by the researcher [Figure 2]. These 5–10 min neutral sessions served both to reset the human’s experience and to provide multiple same-day comparative baselines as features of emotions that were relatively non-stationary [8,65,66]. While some canine subjects rested during the human neutral subsessions, the official baseline for the dog occurred during a separate set of 5–10 min periods, during which they wore the physiological equipment and came to a natural rest state (i.e., they relaxed, crouching with head down or otherwise lying down fully) in the presence of their human partner and the researcher. In keeping with the field best practices, the evaluation methods were mixed, including physiological data collection, human subject surveys, and behavior coding [12,39,67,68,69,70,71]. All the test procedures were approved by the NC State University Institutional Review Board and the Institutional Animal Care and Use Committee.

As previously discussed, this study focused on both human and canine subjects’ responses to the interaction. Therefore, both participants wore physiological data-collection equipment in this pilot study. The SySy prototype used included (i) the custom chest patch, custom wrist band, and Empatica E4 for humans, along with (ii) the custom smart collar and custom harness systems for dogs [67]. Each device had been introduced by our research group previously [16,17,18]. These devices were selected for the biological signals acquired, their high sampling rates, their stability during movement, their positive ergonomic profiles, and their relative ease of use. Together, they make up the first synchronized system for interspecies interaction measurement. As part of this human–canine interaction research setup, and for post hoc behavioral coding, two or more smartphone video cameras were used to capture all angles of the research space during the interaction and neutral subsessions.

### 3.2. Analysis Design

#### 3.2.1. Epoch Selection

Interested in the internal dynamics of a CAI session, we first selected a repeatable time period upon which to focus our analysis. We used the information available in the literature [69,73,74] to determine the time intervals of interest for each collected signal and to select the most appropriate epoch length to track the desired changes in physical phenomena across signals. These existing works showcase that for several relevant affective measures, phenomena changes can be reasonably measured on the 5 s-to-30 s-to-1 min timescales, and several papers in the canine literature have 5 s-to-15 s-to-5 min sliced time frames [69,75,76,77,78]. As such, we selected 10 s epochs to capture the fastest changes (e.g., arousal via inertial measurement units), though we recognize (a) that significant changes in heart rate variability were likely to have occurred somewhat slower than movement activity fluctuations, and (b) that other metrics, like skin temperature, were likely even slower. However, this standardization across metrics was necessary for our proposed analytical approach, not uncommon in the scientific literature, and it still reflected appropriate changes across each metric.

#### 3.2.2. Behavioral Coding Approach

For human–animal interactions, one of the best, validated behavior coding paradigms is observation of human–animal interaction for research (OHAIRE), which provides a more objective rubric for dyadic assessment [57]. Using one–zero interval sampling, this schema tracks facial, verbal, and other physical indicators from each interactant and from the interaction as a whole before applying standard comparative statistics. Even though this tool was specifically created and is often used for evaluating HAIs, there exists incredible variability in the behavior coding methods and there is little consensus on which coding schema is most appropriate for assessing psychophysiological states in CAIs [59,79,80,81]. Other approaches include behavior counting, which begins with determining a time point, emotional state behaviors of interest, and how they will be analytically interpreted [82]. These behaviors and the time points or time ranges at which they occur are then demarcated in software tools, such as BORIS or ELAN, spreadsheets, such as Microsoft Excel, and/or hand-written notes, before general analysis [83,84]. Another approach, referred to as qualitative behavioral analysis, has strong support in the social sciences and involves the integration of a human’s holistic perception of a subject, to produce descriptors like “relaxed” or “frustrated” [82,85,86]. In other words, if behavior counting can be understood as a quasi-objective observational approach, qualitative behavioral analysis is well described as a quasi-subjective perceptive approach.

Our behavior coding approach—herein referred to as psychophysiological state assignment (PPSA)—is a quasi-subjective approach similar to qualitative behavioral analysis, and borrows several elements from the OHAIRE approach as well. It is informed by extensive evaluation of the CAI literature’s coding schema, to isolate the reliable indicative behaviors of affective and affiliative states for each species involved. PPSA, then, involves perceptive coding of each interactant into positive, neutral, or negative psychophysiological states for successive, non-overlapping 10 s epochs throughout the session. To minimize bias and maximize consistency, this coding was performed by three raters, two of whom were previously fully trained in the OHAIRE system [80,87,88,89]. Using Cohen’s Kappa value as a measure of inter-rater reliability in post hoc video coding, the three raters were above the common 80% agreement standard in human–animal interaction studies, scoring 89.9% and 95.7% for humans and for canines, respectively [57,58]. PPSA preserves the temporal benefits of qualitative behavioral analysis and allows raters to use any composition of descriptors to inform assignment to one of the three possible psychophysiological states. These assignments, in turn, can be represented as computer manipulatable, numerical variables: −1 for negative states, 0 for neutral states, and 1 for positive states. It is important to note that these state labels are meant to represent clear regions along a spectrum from negative to positive psychophysiological states, whereas normal qualitative behavioral analysis labels are not necessarily similarly inter-related. It is also important to note that our and other researchers’ interpretations of behavior were limited, and that disambiguating between subjects’ true states and consensus views on what observed behaviors indicate was beyond the scope of this study. As for the affective state, psychological surveys before and after subsessions are considered to be gold-standard ground truths. In the meantime, this PPSA behavior coding approach served as a good, semi-continuous ground truth for the duration of interactions and for non-conversant canine subjects. Subsequent analysis of the behavior coding data utilized basic statistical averages and simple percentages with appropriate exclusion of indeterminate epochs.

#### 3.2.3. Survey Selection and Analysis

Our literature search provided several options for relevant survey instruments to be considered (for an exhaustive list, see Appendix 1 in [90]). Six surveys were used in this study for primary comparison and as ground truth options for certain physiological data collected: (i) Canine Behavioral Assessment & Research Questionnaire (C-BARQ); (ii) Monash Dog Owner Relationship Scale (MDORS); (iii) Self-Assessment Mannikin (SAM); (iv) Positive and Negative Affect Schedule-Short Form (PANAS-SF); (v) Human Ergonomics; and (vi) Canine Ergonomics. The non-ergonomic short-term surveys, SAM and PANAS, were completed by hand before, between, and after the experimental day subsessions; they are capable of measuring short-term fluctuations in valence, arousal, positive affect, and negative affect or anxiety [91,92]. These four parameters from these two instruments were the closest to and best measures of our desired conceptualization of psychophysiological states that (i) were also available as relatively brief psychological surveys, (ii) complemented the affective inferences to be made from our physiological data [64], and (iii) were robustly validated in the literature for our and other use cases [12,93,94,95,96,97]. The human and canine surveys for ergonomics were internally developed and were completed at the very end of the experiment by the interacting human subject. The C-BARQ and MDORS long-term surveys were completed at the human subject’s leisure outside of the experiment. These targeted in MDORS a common measure of human–canine relationships and in C-BARQ a standard evaluation of the dog’s general behavior [32,98,99]. It is important to note that all human subjects were required to complete MDORS, but only the dog’s primary owner completed C-BARQ. In addition to following the survey instrument developers’ recommendations, the survey data analysis used basic average statistics as well as the Wilcoxon signed-rank test for general data comparisons and repeated measures data. We considered a 2-sided *p*-value of <0.05 to be statistically significant. To keep this study more concise, the ergonomics surveys and the C-BARQ behavioral survey results will be analyzed and presented in a future publication.

### 3.3. Physiological Data Analysis

#### 3.3.1. Signal Selection and Calculation

All physiological signal metrics were selected upon extensive review of human and canine psychophysiology in response to interaction and indicative of affective states. Using the selected epoch time frames and the classic psychophysiological theoretical framework, we took the raw physiological devices’ data and completed a preprocessing step that included an initial data check and removal of outliers. We then filtered each signal, using bandpass Butterworth filters, we completed a normalization step, and we achieved temporal synchronization across the multimodal device signals and with the behavior coding output [36]. The second core analytical step included two forms of metric extraction: average metric by epoch (ME) and rolling window average by epoch (RE). From the accelerometer signal (also referred to as the activity signal or inertial measurement unit (IMU)), we directly calculated the average, minimum, and maximum acceleration by epoch along each spatial axis, before calculating the mean amplitude deviation (MAD) by axis and the integral modulus of acceleration (IMA) across dimensions [35,100,101,102,103,104,105,106,107]. Similar IMU metrics to those described above were also supported for analysis of canine activity [108,109,110]. From the ECG signal, we used ECG waveform R peaks to extract the interbeat interval (IBI), using the “Pyphysio” toolbox in Python 3.7 via Google Colaboratory Jupyter notebooks [111]. With IBI serving as the basis for all the other ECG metrics, we then determined heart rate (HR) and three additional heart rate variability (HRV) metrics in the time domain. These included the standard deviation of the IBI of normal sinus beats (SDNN), the root mean square of successive differences between normal heartbeats (RMSSD), and the quotient of SDNN and RMSSD [38,75]. Briefly, RMSSD estimates “vagally mediated changes” in HR while SDNN tracks both parasympathetic and sympathetic nervous system activity contributions to the recorded HR [112]. As noted, IBI and HR extraction is standard for ECG analysis, and the three HRV metrics were well supported for both human and canine evaluation of valence, stress, and other psychophysiological constituent states [20,32,37,38,75,76,77,78,112,113]. From the skin temperature (ST) signal collected by the Empatica E4, we simply determined the average ST value by epoch [39,114]. From the electrodermal activity (EDA) signal, we extracted the average and maximum EDA values to characterize the combined galvanic skin response. We also ran this signal through the developer’s EDA Explorer online platform, to remove artifacts, to detect the phasic skin conductance response (SCR) peaks for short-term stimuli, and to differentiate the tonic skin conductance level (SCL) long-term baseline [108,115,116]. The EDA analysis in this paper focused only on the SCR short-term stimuli responses. RE—the rolling window metric extraction—calculates the same metrics from the same preprocessed signals as the ME approach but, rather than a sequential averaging by 10 s epoch, it uses a centered, 60 s, rolling window to produce a 10 Hz output signal (e.g., from a 200 Hz chest ECG signal, RE produces a 10 Hz average heart rate signal). Though we extracted a 10 Hz RE signal for all of our metrics across all 5 devices, the RE output is expressly used herein for correlational analyses of synchrony only. The selected output frequency of 10 Hz was based on the human and canine torso signals held in common (i.e., chest ECG and chest IMU on both subjects). While all signals or metrics were used and investigated throughout the analysis, for spatial economy we present a meaningful subsample of signals in this paper.

ME signals were processed as appropriate to produce the summary tables and heatmaps displayed throughout. For each experimental session, we also calculated the difference between epochs by metric, and we marked the increase or decrease of each metric over the entire session. Then, referring to our literature review, we assigned a direct or inverse relationship from that metric to the expected effect on psychophysiological states, and we coded the epochs throughout the session for their positive or negative contributions to said states. All metrics were also associated with and grouped according to valence (herein also called stress) or arousal. Afterwards, these heatmaps were inspected visually for vertical and horizontal patterning.

#### 3.3.2. Data Analysis Part 1: Overall Methods and Interpretation

Again, the products of the aforementioned metric extraction or ME step contained the biometrics averaged over each epoch. We read all of these ME results from the various devices assembled under the SySy prototype, and we synthesized them by performing 6 epoch averages at the beginning, middle, and end of each subsession, representing key minutes from the dyadic interaction. For overall reporting of physiological data by signal, we used average test statistics and the Wilcoxon signed-rank test to compare between session types; we used the Pearson correlation test statistic for comparisons between multimodal data averages across interaction subsessions [19,20,75].

Given the large number of physiological signals collected from each dyad, there was some nuance to their individual and joint interpretation. Most reported sources find that increases in HR, EDA (e.g., skin conductance responses), and ST are generally understood to indicate elevated arousal in humans [19,63,67,117]. Additionally, increases in HRV time domain metrics (specifically, SDNN and RMSSD increases) indicate a decrease in stress and potentially more positive states/emotions [19,20,38,118,119]. For interpretation of canine physiological signals, increased HR often indicates higher arousal and increased HRV also indicates more positive canine states [29,75,76,77,78,113]. For both species, we assume that sustained increases in average movement in three dimensions over a given epoch of time indicate more arousal and, thus, less calm states for that subject. We followed these broad field guidelines for interpretation of our results, but note that further independent validation of these directionalities for each species was beyond the scope of this work as there was no “one-to-one relationship between emotional changes and autonomic activation” [120]. Additionally, the debate surrounding a complete psychophysiological theory of emotional states and their interpretation for humans, not to mention animals, is ongoing [19,52,63,121]. Lastly, we acknowledge that psychological surveys and our behavior coding approach, by design, produce state-based outcomes while the physiological approaches can only produce directional outcomes in comparison to previous time periods’ signals.

#### 3.3.3. Synchrony Methods

The wearable systems were located on both human wrists and on the human chest as well as on the canine’s torso and neck. As such, we only considered the torso systems, representing the signals shared between species, for synchrony investigations of bonding. While of potential interest for exploring previously unknown inter-relations and for identifying relevant movements, like dog petting, for example, the data from the other subsystems either had no direct correlate in the opposite dyadic counterpart’s subsystems or would necessarily have resulted in spurious data (i.e., it was likely not valid to correlate human hand motion to dog neck motion.) Additionally, psychophysiological measures closer to the center of mass are generally understood to be less prone to movement artifacts [19,100]. Using an 18-epoch (i.e., 3 min) RE slice taken from the middle of each interaction subsession, we used two approaches to determine interactional synchrony as a proxy for bonding. First, the overall Pearson’s correlation for our three key ECG metrics (e.g., HR, SDNN, and RMSSD) and for one key activity metric (e.g., IMA) was calculated [122,123]. We further tested the metrics’ interspecies interaction via the dynamic time-warping methodology, to track these key time series’ data alignment in general and when assuming temporal asynchrony [123,124].

## 4. Results

We were able to successfully deploy wearable physiological measurement systems on both human and canine subjects simultaneously and continuously as they interacted. Analysis of this pilot data is presented in this section and attempts to answer the aforementioned questions of interest to the AAI, HAI, and CAI fields.

### 4.1. General Survey Responses

**Figure 3:**
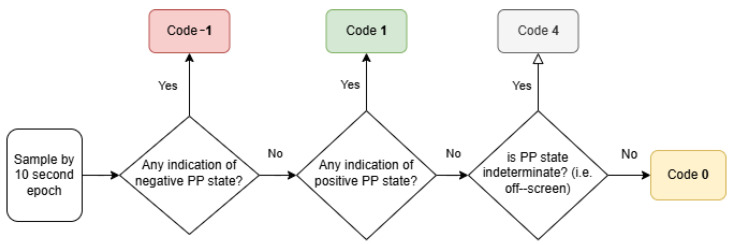
PPSA behavior coding rules flowchart. Epochs are coded based on the majority of perceived states within the epoch.

For the survey responses, we investigated the time and type dependencies of the valence and arousal outputs from the SAM and the positive and negative affect outputs from the PANAS. The four survey scales were taken during interstitial experimental periods, meaning there was no survey before the baseline session. For positive or negative affect (i.e., “PA” and “NA”, respectively) larger numbers indicate more positive or more negative affect [Table 3]. For the SAM valence and arousal scores (i.e., “V” and “A”, respectively), larger values indicate more unhappiness and more calmness, respectively. Where appropriate (i.e., excluding surveys from 2 of the 22 sessions for participants missing, incomplete, incorrectly filled out, or otherwise spoiled survey data) we ran the non-parametric Wilcoxon signed-rank test, using the self-same function from the SciPy library to compare outcomes for neutral-type to interaction-type sessions [75,125]. For individual subsessions, some clear patterns emerged. SAM arousal consistently increased after an interaction session, on average, compared to the neutral sessions. A similar pattern can be seen in the PANAS positive affect, which reliably increased, on average, with the interaction sessions. The SAM valence results by subsession were more variable, but the PANAS negative affect indicated a reliable decrease after interaction sessions. For all neutral vs. interaction session types, we saw a significant difference in SAM arousal (*p* = 0.043) and SAM valence (*p* = 0.0002). Looking at the PANAS dimensions, the full group of subjects saw a significant difference in positive affect (*p* = 0.0003), with no major difference in negative affect observable in this study (Figure 3). Overall, our study group saw significant self-reported state changes indicating more arousal, more positive valence, and more positive affect. Though decreases in negative affect were common, no significant change occurred across subjects with canine interaction. While these survey results are preliminary, they are promising and make intuitive sense for CAIs.

### 4.2. General Behavioral Coding Outcomes

For camera-based behavior coding, we focused on the percentages of each interaction-type session spent in each psychophysiological state by animal–human pairing, excluding periods where either subject was off screen as indeterminate. This was done as the dogs were not necessarily resting during the neutral-type sessions (i.e., outside of the interaction space, pacing, watching, and, otherwise, waiting,) and the neutral session human psychophysiological state codes by epoch were unvarying (as these subjects were instructed to sit and listen to music, read, etc.) Of course, this eliminated any meaningful comparison of behavior coding scores between session types, though it does lend some credence to the significant differences seen between session types for the survey results. More simply, the interaction sessions were characterized by all three psychophysiological states, for both participants, whereas the neutral session results were completely neutral, for the human, by design. Beyond these observations, the first notable overall outcome was the high number of neutral ratings by interaction session (i.e., typically over 60% of on-screen time). This indicates that neither interactant was visibly or audibly in a positive state for most of the CAI sessions within our study [Table 4]. As expected, negative ratings accounted for a vanishingly small percentage of the canine and human behavior codes. Characterizing the majority of positively coded epochs, the dogs generally displayed more affiliative and affective behaviors in goal-oriented interactions (i.e., in order to solicit attention or treats). While positive codes for either interactant seemed generally higher for some pairings than others, no other clear patterning emerged across all subjects.

We also applied an “exclusive nor” logic gate to the behaviorally coded scores by epoch, to investigate the synchrony between dyad members, showing the percentage of the interaction session for which the dyad had the same one of the three psychophysiological state codes between the species. Epochs with either party off-screen were excluded, and were also the impetus for this novel form of synchrony analysis. Across the board, pairs spent much of the session time in the same psychophysiological state. This was likely due to the high percentage of neutral ratings for both parties in most interactions. Looking at successive sessions, there appeared to be no consistent synchrony patterns as the dyads had more situational contact.

### 4.3. General Physiological Data Outcomes

For the IMU data acquired by the SySy custom smart collar device on small and large dogs, the placement of the smart collar was not found to give results that were meaningfully different from the torso-located harness IMU. The smart collar did tend towards more noise and exogenous movement, as it was attached to loose-fitting collars. Therefore, we only present the torso IMU signal acquired by the SySy harness device. While the smart collar also collected ambient environmental measures beyond physical activity, we decided to focus on dyadic interaction, and we kept back the analyzing of the effect of the environment, as being beyond the scope of this paper, thereby reserving it for a future work.

Table 5 reports our average results across three time points within interaction or neutral subsessions for target signals across our wearable device system. Upon visual inspection, the human and canine HR and HRV results do not indicate clear patterning across subjects or session types at this scale of analysis. For the cluster of activity data represented in the last four columns of the table, it appears that left-wrist movement occurred much less than chest or right-wrist movement, which concords with the fact that all the included human subjects were right-hand dominant. Furthermore, within the subsession groupings, each IMA source seemed to remain relatively stable, though the differences between the neutral and interaction sessions were not statistically significant.

Table 4 also reports the Wilcoxon signed-rank probability that there was a significant difference between the neutral and interaction session types for each signal presented. Of note, the canine harness HR signal, the human right-wrist ST signal, the human EDA mean, and the EDA max scores differed significantly across the subjects, in this respect. As noted previously, the canine subjects were removed from their experimental interactant during the neutral subsessions and escorted by a researcher during this time. While the dogs were not expected to also engage in neutral behavior and were free to do anything, from interacting with the human to resting quietly during these subsessions, these comparison results may indicate that focused one-on-one interaction is meaningfully distinct from regular real-life activity in this context for a canine HR. If true, this could positively indicate the idea of at-leisure breaks being recuperative or, at minimum, positively different for therapy dogs while at work. As the ST signals indicate, the localized temperatures did appear to rise across the subjects as the experimental sessions progressed, when placed on the right hand of all the subjects. This was likely due to the increased physical activity with their dog interactant. The strong difference between the neutral and interaction sessions may have been due to the relatively low baseline temperatures initially observed, on average. Generally, the EDA average amplitude by epoch and the EDA maximum amplitude by epoch—both arousal indicators—seemed to reliably and significantly increase during the interaction sessions, as expected. This comports well with the survey self-report findings of increased arousal after interaction sessions across the subjects, as previously discussed. It is worth noting here that across our analyses, and in keeping with other studies, EDA seemed to be one of the more reliable and responsive differentiators between neutral and interaction sessions for the human participants throughout the experiment. Upon further analysis, other signals may prove to have been individually predictive or to have also correlated with overall affective states, but the EDA metrics appear to have had clear and multifaceted support between session types.

### 4.4. Multimodal Composite Results

**Figure 4:**
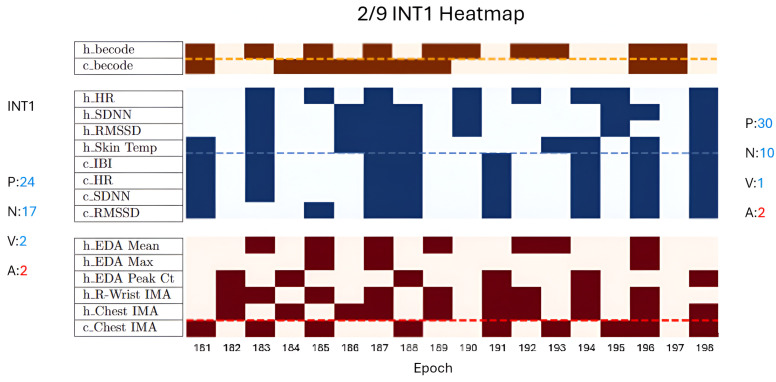
CAI subsession heatmap example with physiological, behavioral, and survey data. P = PANAS positive affect; N = PANAS negative affect; V = SAM valence; A = SAM arousal; INT1 = interaction session 1; h_ = human; c_ = canine; R-Wrist = right wrist; becode = behavior coding; HR = heart rate; IBI = interbeat interval; SDNN = standard deviation of NN intervals; RMSSD = root mean square of successive differences between heartbeats; SKIN TEMP = skin temperature; EDA Mean = average electrodermal activity by epoch; EDA Max = maximum electrodermal activity by epoch; EDA PEAK Ct = number of peaks in epoch of electrodermal activity; IMA = integral modulus of acceleration. The dashed lines separate human and canine metrics.

To derive composite results, we took an in-depth look into some CAI sessions to see how the patterning of metrics contributed to the overall outcomes. As noted before, this was done by taking the ME outputs and tracking whether they increased or decreased from epoch to epoch. Then, using careful directional indicators from the literature, we created heatmaps that represented the 3 min segment directionality of the available valence and arousal dimensions in a bonded individual, shown in Figure 4 [52,77,95,118]. In this heatmap, the blue section represents metrics correlated negatively with stress while the red represents positive arousal metrics; the canine metrics are below the dashed line on each dimension’s chart. A solid color indicates an increase while the absence of color (e.g., off-white tinted red or blue) indicates a decrease in the psychophysiological state metric for that epoch. Within each section, a dotted line separates the human signals from the canine signals, as well as further “h_” and “c_” prefix demarcations for human-sourced and canine-sourced signals, respectively. The signal type (i.e., physiological, survey, and behavior coding) naming conventions followed the common abbreviations previously indicated in this paper. For these charts, blue blocks thus represent psychophysiological state increases along the valence dimension and red blocks indicate psychophysiological state increases along the arousal dimension.

Unexpectedly, we see no clear overall patterning for each subsession by type across subjects. However, we note that the neutral session’s human EDA mean and EDA max metrics decreased noticeably for most of the subjects when compared to the interaction sessions. This reflects the significant change in the surveyed arousal score and the strength of EDA as an arousal metric. For canines, the neutral, baseline, and post-line session metrics do not reflect resting. However, looking vertically, the canine valence epochs tend to show a higher degree of coherence across the signals and metrics (i.e., all increased or all decreased). These representative visual examples of the patterning within sessions juxtaposed to the survey outcomes are uniquely made available to researchers by the continuous, multimodal wearable system coupled with our experimental approach, and they allow for multimodal output alignment. Taken altogether, this heatmap representation, showcased in Figure 4, indicates to researchers the dynamics of the session or session slice across behavior coding and physiological signals as well as the survey outcomes that bracket the interaction. It also allows for fast visual inspection of vertical bands for signal coherence or horizontal bands for expected macro-trends in certain signals (e.g., EDA signals consistently decreasing during a neutral session, canine RMSSD indicating negative experience, etc.) [75,76,116,126].

### 4.5. Physiological Data Snapshot

**Figure 5:**
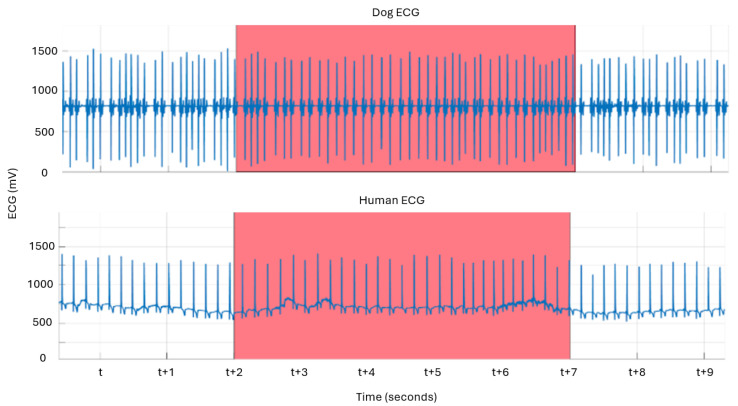
ECG signal CAIs with highlighted events [72].

The Figure 5 raw signal plot is a representative glimpse of the original ECG data for humans and canine subjects that were simultaneously produced by our multimodal system during the experiment. The brackets are marked with colored regions, to show where the metrics might deserve inspection since the bracket entered was a time of interesting activity. In our approach, this is useful for several reasons. Firstly, it highlights basic, enduring differences between the species, like a canine heart rate being faster than a human’s, on the whole, affecting the signal processing (e.g., sampling rate, filtering, and amplification) parameters. Secondly, indications from other data streams could prompt us to look at the raw and derived signals for that time period (e.g., during behavior coding, a visually observed strong negative reaction in the dog vs. the giving/receiving of a treat), for further inspection/analysis.

### 4.6. Behavioral Coding Subset

Like the heatmap, a synchrony table provides an interesting multimodal snapshot of the experimental data from this study. Though it is challenging to present the data from all 34 interaction sessions, the table in Table 6 showcases two interaction subsessions, each from three humans in total as they interacted with the same dog. The arousal, valence, positive affect, and negative affect survey scores do not show clear patterning based on bonding here. However, lower MDORS scores indicate a stronger bond and, as expected, H1–C1’s owner proved to be the most bonded to the dog by the survey result, and H2—a friend of the dog—was less bonded, while H3—a stranger to C1—was the least bonded. These differences and this ordering are also directly reflected in the behavior-coded amount of time each pairing spent in positive states. For both interactions presented, H1 and C1 each spent much more time in positive states than the moderately bonded pairing of H2 and C1, or the weakly bonded pairing of H3 and C1. This resulted in the MDORS survey score and amount of time each member of the interacting dyad spent in positive states being the measures that most closely track with the expected level of bonding. A potential counter-indicator of bonding was the presence of negatively coded epochs for the canine. While there were relatively few negative states coded throughout the entire pilot experiment (1.6%), all of these occurred in interaction sessions between a dog and a non-bonded human (i.e., when the canine was not interacting with his owner). Surprisingly, behaviorally coded epochs spent in the same state appear to be much lower in the bonded pair when compared to moderately and weakly bonded pairings. This unexpected result actually follows from the fact that in most cases the majority of an interaction session was spent in neutral states, leading to a very high same-state % result in the non-bonded interaction subsessions. In the bonded pairings, the dog and human matched in some epochs but largely differed, due to the nuances of certain interaction behavior sequences. For example, in some instances, the human would display positive affective and affiliative behaviors while the dog consumed a treat, whereas the dog displayed these behaviors as the human was presenting the treat, leading to mismatched positively coded epochs and potentially pointing to the pleasure cycle theory of behavioral response in dogs [29]. Additionally, with their owners the canines tended to exceed the number of affective displays observed in the human, while with non-owners they displayed far fewer affectively positive displays than the human. While these behavioral indications are an initially promising way to measure interspecies bonding, studies with larger sample sizes and further replication of these results are required, to confirm these findings. Turning to synchrony between the selected physiological signals, our Pearson correlation results seem to indicate that the interspecies paired signals were not significantly different from each other, but they do not show other clear patterning by signal type or by bond level. We also used dynamic time warping and minimum signal distance analyses to further approximate the level of bonding. Like the previous correlations, the dynamic time warping results show no clear patterning across subjects other than heart rate signal results spanning much larger path distances than the other key signal types evaluated by this method. This was likely due to the significantly higher canine HR when compared to humans, and may also factor in certain differences in HRV between the two interactants. These two sets of correlation results strongly hint at further exploration being needed of physiological synchronization between participants as a measure of bonding.

### 4.7. Multimodal Correlation Matrix

**Figure 6:**
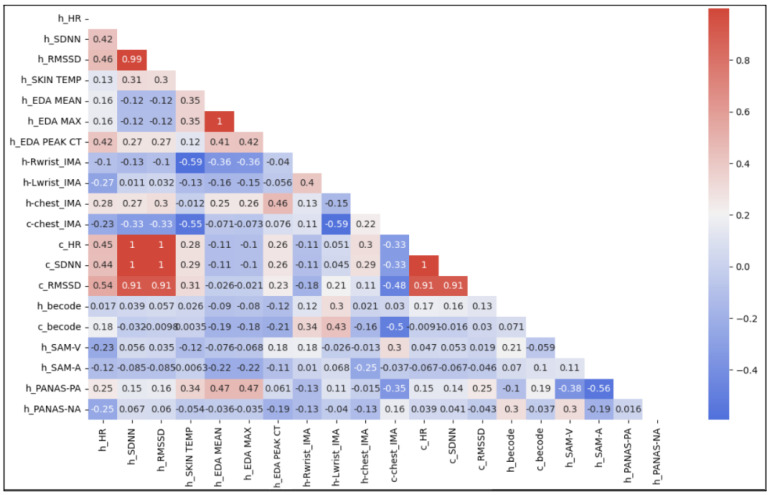
Correlation matrix for interaction data [72]. h_ = human; c_ = canine; PANAS-PA = positive affect; PANAS-NA = negative affect; SAM-V = valence; SAM-A = arousal; INT1 = interaction session 1; Rwrist = right wrist; Lwrist = left wrist; becode = behavior coding; HR = heart rate; SDNN = standard deviation of NN intervals; RMSSD = root mean square of successive differences between heartbeats; SKIN TEMP = skin temperature; EDA Mean = average electrodermal activity by epoch; EDA Max = maximum electrodermal activity by epoch; EDA PEAK CT = number of peaks in epoch of electrodermal activity; IMA = integral modulus of acceleration.

We computed an individual correlation matrix across all the subjects and the full multimodal dataset as an exploratory analytical approach. Focusing only on sessions where both species of subjects interacted (i.e., no neutral sessions), we took the average of the middle minute of data for each behavior coding and physiological signal, as well as the post-interaction survey scores, to populate this matrix. This resulted in a comprehensive overlay of signal interactions across the experimental sessions and subjects. Of considerable note, time series HRV indicators had strong positive associations within species, as expected, but also across species. These are already considered to be some of the best indicators of psychophysiological states and could serve as a reliable indicator of interspecies interaction or bond quality in future work. The integral modulus of acceleration (IMA) showed some moderate correlations in a few signal types. For the human right wrist, the IMA was associated with skin temperature, possibly indicating a heating effect of additional human movement, likely due to stroking, brushing, and other interaction-specific activities. The canine chest IMA was also moderately associated with human skin temperature for reasons that are less intuitively clear. This IMA variant also associated moderately with behavior coding for the canine and with the RMSSD HRV metric. That finding may indicate that the rater’s perception of the canine state may have been somewhat influenced by the dog’s movement, and it potentially reaffirms previous findings that RMSSD is a reliable state indicator in dogs [75,76]. Lastly, though most other correlations between the multimodal signals from this experiment were weak, the human self-report arousal scale was moderately associated with the positive affect self-report scale. This relationship was echoed in our other analyses, and it potentially indicates that a contributing factor in the overall positive affect in the humans was the level of arousal inspired by interaction with the dog (Figure 6).

## 5. Discussions and Future Work

Psychophysiological states can be a proxy for quality of life, and wearable sensor systems have shown some promise for quantifying these states, though they have not been sufficiently developed for or deployed in joint human–canine research in the past. This pilot study advances our goal to use a synchronized wearable device system to quantify multiple, interspecies CAI subjects, aspects of their bond, and their psychophysiological states during repeated interactions. As such, there are several topics requiring further discussion.

Though the behavior coding outputs, the EDA signals, SAM arousal, SAM valence, and PANAS PA showed significant differences between neutral and interaction subsessions by design, our results did not clearly and consistently indicate such differences across data modality and metric type. This conforms to most other CAI results with or without baseline sessions or control groups. A potential reason is that, while a canine interaction is expected to confer positive benefits above and beyond baseline, the activities typically selected for control groups and neutral sessions (i.e., meditation, light reading, etc.) also provide a different kind of potential benefit to participants. One way to disambiguate this is to induce positive, negative, and neutral states in multiple subjects (traditionally by making them view emotional movie clips) before proceeding with a CAI session and analyzing the metric deltas.

Overall, our impression is that the evidence of bonding is most apparent in behavior coding, since not all psychophysiological states are created or displayed equally between humans and dogs. While this somewhat limits the generalizability of behavior coding assignments between participants, it does open the door to various kinds of streak matching. As opposed to investigating preset slices of multimodal data interaction, our and others’ future work could search for streaks of sequentially displayed states to compare within and across subjects or species. Beyond epoch streaks of coded emotional states, future investigations could be optimized for other parameters, such as “proximity”, where both subjects are close to or in contact with each other, or “mutual attention”, where both subjects focus on and orient towards each other.

Though the sessions were designated as interaction periods, the more granular dynamics, as revealed by behavior coding, painted a more complex picture, where the CAI itself was not responsible for all the observed states. For example, the humans often became moderately frustrated with the dog constantly leaving them during the interaction rather than with the canine interaction itself. In fact, this was actually the source of all the negatively coded human epochs, whereas all the canine negative codes were generated in response to human actions. In other cases, the human might display positive affective behaviors due to their own internal mental state, when the dog was not nearby or when the human was focusing on something else (e.g., looking around the room and singing to self).

Although unintended, the behaviorally coded differentials in the total percent of epochs on screen between the interacting humans and canines sans leash may have served as a partial proximity metric and, thus, as an additional indicator of the bond. That is, if the human remained on screen for behavior coding but the canine had run away, and stayed away, for a significant portion of the interaction time, this could have provided some additional information about the character and quality of the interaction session and the pair’s bond, given the appropriate context and caveats. It could also have indicated the interaction choices of the human (i.e., offering treats more often is likely to keep the dog nearby and on screen), the activity level, the exploratory nature, and the other temperamental features of the participating canine (i.e., more active dogs may exit camera shot more often). However, we could not go further with this analysis, as participants were free to choose whether or not to leash the dog during this pilot test.

Additionally, while our results are somewhat different in form, we observed non-alignment with another CAI study (the only one available in the literature, to the best of the authors’ knowledge) conducting a correlational bond analysis [32]. That work found that individuals with a weaker bond to their dog, as determined by MDORS, seemed to have some statistically significant correlation between HRV and oxytocin measures. Herein, though no biochemical analyte was analyzed, the results of our work seem to indicate that HRV measures between interactants have no clear patterning as the theoretical strength of the bond increases. This warrants deeper investigation and a larger analysis beyond a pilot study.

For our interspecies synchrony data, we noted that the behavior coding results from the bonded pairs seemed to be less alike than for the unbonded pairings. This result is surprising as, in keeping with the core concept of synchrony, individuals are expected to closely match or reflect their bonded counterparts. Our results indicate that some aspects and assumptions of the synchrony hypothesis may not hold across data collection methodologies or contexts, especially considering that the majority of interaction session epochs were neutral when coded. This means that the type of synchrony matters (i.e., temporally synchronized positive states in strongly bonded pairs) and could potentially add more detail to future bond/dyadic interaction analyses (e.g., moderately synchronized positive states vs. highly synchronized negative states.) Interestingly, the dogs only presented negatively coded epochs in interactions with unbonded individuals. Since the humans almost universally showcased neutral-to-positive states (in parallel with this study’s surveys and the general literature), the presence of some negative canine codes could be a very useful metric of the impact of interaction on the therapy animal [2]. This is especially true considering that the PPSA behavior coding approach captures the dynamics, responses, and outcomes of the canine with higher granularity than in physiological averaging or survey pre-/post-analyses alone.

This leads directly into one limitation with the PPSA behavior coding approach. The PPSA rules bias epochs towards neutral scoring and can also drown out non-neutral results. The former bias occurs because 5 s of neutral accompanied by 5 s of positive or negative is always coded as neutral. Drowning out, on the other hand, may occur if a continuous period of 10 or fewer seconds of positive display, for example, just happens to be split evenly between two epochs otherwise characterized by neutral display. In this case, both epochs would be coded neutral. More generally, PPSA relies heavily on the epoch start time and epoch size selected by the researchers, and, once set, it has the aforementioned impacts on the resulting analyses. By comparison, surveys are even more inflexible than PPSA behavior coding (i.e., only administered before or after an interaction) while continuous physiological data is more easily adapted to epoch start and size alterations.

Herein, we attempt to not only present the data from each methodology independently but also to look across multimodal data streams in order to identify psychophysiological states. On this latter point, [12,19,67,68] go out of their way to encourage future researchers to use multiple evaluative methods to reduce bias and increase convergent validity—especially via the merging of several “mutually relevant...physiological measurements” for improved and/or disambiguated effective detection. However, this type of work is still nascent in human–canine interaction studies and also in animal psychophysiology in general. This presents the many challenges of experimentation and interpretation that are noted throughout this paper. One worth mentioning is the challenge of the core psychophysiological assumption that distinct states can be reliably indicated by specific directional signals and derived metrics. Although the pilot data in this paper were interpreted according to traditional psychophysiology techniques, this study was also designed to collect data in a way that permits interrogations of this cluster of alternate approaches. Our future work will pursue this via inclusion of contextual/environmental data and via individualized subject-by-subject analyses for both species. Including an environmental context may help to better interpret interaction results and enable high-resolution indoor vs. outdoor comparisons; it could help researchers study the connection between weather and behavior/physiology in both subjects; and it could also enable more detailed input feature vectors for machine learning and for other causal attribution efforts.

Another challenge was the dependence of the performed analysis on previously established psychophysiological directional indicators, inherently due to the aim of this study to explore correlations and predictions. While these directional indicators were based on systematic reviews of the literature, the precise relationships between certain measurements and between human or canine states were neither fully settled nor correctly assumed to be consistently one-to-one [120]. The indicators for the canine perspective were also far more sparse and unsettled than those on the human side of the equation. In a sense, indicating that certain metrics increased and decreased by epoch is a more conservative interpretation of these results until direct correlations to emotional states are fully established by other psychophysiological and neuroscientific research. To handle the non-stationarity of the data, increases and decreases by epoch by subsession were used as psychophysiological state indicators, but an alternative approach could be to establish baseline values from the same day and track the values as they cross above and below these averages, to identify psychophysiological states [8,65]. While this second approach is potentially more individualized and is certainly possible, with a wearable system acquiring data continuously and the pilot test protocol implemented, it involves a fair amount of arbitrary decision making and/or novel statistical depth that was beyond the scope of this paper [77,127]. Future longitudinal studies will add individual subject and state emotional inducements to serve as the baselines for all analyses conducted on the same day, despite the potential for downstream effects on the CAIs.

In this work, the physiological data was primarily viewed through the lens of relative increases and decreases of selected metrics by epoch. This approach was appropriate for the scope of this work, as we did not set baselines for each subject with repeated psychophysiological state emotional inducements, and as the emotional metrics were non-stationary across the experimental days [8,65,127]. This must be kept in mind when comparing physiological increase/decrease outcomes to behavior-coded data—which represent continuous rater psychophysiological state perception assignments—and to survey data—which represent subject self-scoring of independent psychophysiological state dimensions pre-/post-interaction. An important distinction between this work and other research in the literature is that herein we used manual analyses to interrogate the nature of the CAI sessions. Most recent emotion detection analysis has relied on machine learning methods such as random forest, support vector machine, or regression in combination with other statistical tools to label and classify data segments by predetermined emotional category [37,38,39,76,95,128,129,130,131]. While this remains a future possibility for our epoch dataset, using the psychophysiological state behavior codes as labels, we wanted to build our investigation of the data from the ground up, considering the project’s novelty, the difficulty of defining emotion state boundaries, and the relatively small sample size/amount of data collected in this pilot effort.

On the point of better dyadic emotional state detection, we suspect that further analysis epoch-by-epoch between the behavior-coded outputs and continuous physiological data as well as digging deeper into individual responses to the interactions will prove more fruitful than aggregated outcomes. However, the work discussed herein does accomplish our proximal goals of deploying synchronous interspecies wearable systems, analyzing continuous data, and generally characterizing CAI internal session dynamics beyond the current pre-/post-state of the art. There is also considerable interest in looking across measurement modalities, to begin to identify patterns and answer questions of emotion coherence. This latter concept speaks to the idea that an experienced emotional stimulus should be represented across all known psychophysiological indicators and that, potentially, the same response is trackable across iterations of similar stimuli and can, thus, be reliably identified. To this end, our preliminary analyses across behavioral, psychological, and physiological modalities indicate that interactions tend to increase measures of positive affect, measures of valence, and measures of arousal when compared to neutral sessions. Though the behavior coding and physiological data contributed to this outcome, they were more ambiguous and require further investigation. For CAI, survey data remains the clearest directional psychophysiological state indicator, as expected. Therefore, our approach and systems can enable many avenues of future work, including but not limited to extended synchrony explorations, psychophysiological state predictive analytics, directional coherence across signals, and both individual and longitudinal emotional state inducement.

## 6. Conclusions

This study was a first attempt to use wearable systems on humans and dogs for continuous and simultaneous physiological and behavioral data collection towards correlational analysis for determining synchrony and bonding in interacting dyads during canine-assisted therapy sessions. We assembled a custom wearable sensor system prototype to perform a pilot study. We were also able to peer into the underlying dynamics of the continuous CAIs that lead to the macro pre-/post-survey results commonly reported in the field. Of particular note, we presented three novel multimodal data representations for potential characterization of CAIs: a subsession heatmap, a synchrony table, and a metric correlation matrix. Lastly, several of our exploratory analyses yielded interesting proof-of-concept results, to inspire future investigations.

In the section titled “Part I: Sensor System Development”, we confirmed that the most common methodological approaches in CAI studies are behavioral coding, biochemical assays, and psychological surveys. As the field turns to more objective approaches, physiological measures such as ECG (especially heart rate/variability derivatives), oxygen saturation, respiratory activity, and blood pressure are becoming more prominent. However, the field still struggles with often ignoring the canine’s perspective, with large variation in study designs accompanied by lack of appropriate controls, and with general measurement method inconsistencies. Following up with a select group of CAI stakeholders, we discovered that many are unsatisfied with the current measurement state of the art, that they desire non-invasive alternatives, and that they suggest inclusion of environmental and other contextual signals, though there is growing consensus around desiring intuitive data formats and positive psychophysiological state indicators. For these respondents, our custom sensor system promises to address the noted issues, and, thus, we further characterized the system, confirming that it could perform well in a typical CAI context (i.e., session duration, distances traversed, etc.) and that it collects high-quality data. Before full experimental deployment, we also comparatively assessed the ergonomics of our synchronized system devices individually and combined, before making any necessary sensor, signal, or packaging adjustments.

Overall, the pilot study presented in the section titled “Part II: Pilot Study and Proof of Concept” confirmed common CAI field results like canine heart rate being significantly higher than humans during interactions, as expected, and humans generally reporting positive-to-neutral outcomes thanks to the interaction. Interrogating the physiological data collected in this study, we found that the electrodermal activity measures were the most meaningfully distinct between the neutral and interaction sessions across the subjects. For the survey data, we saw significant positive changes in subjects’ arousal, emotional valence, and positive affect with canine interaction. Counterintuitively, nearly all the interaction time periods were rated as neutral, with relatively few positive epochs and significantly fewer negatively coded epochs. However, we suspect that this was partially influenced by the chosen coding schema and epoch time period duration. This preponderance of session neutrality also contributed to the moderately high amount of interspecies synchrony observed behaviorally, though the bonded pairs seemed to have lower levels of coded synchrony than expected. While the physiological synchrony results hint at promising associations, the results were not definitive for the four metrics interrogated (i.e., heart rate, heart rate variability (SDNN, RMSSD), and activity (IMA)). Canine surveys were not employed, but the standardized measure we used showed clear bond quality discriminatory power between owner of, friend to, and stranger to a dog. The independent canine results of potential interest are the associations between canine chest integral modulus of acceleration and human skin temperature, canine behavioral coding, and the dog’s own RMSSD heart rate variability. Though moderate, these indicate several potential areas of follow-up investigation on the canine side. Lastly, the dogs only seemed to experience negatively coded epochs with unbonded human interactants as a result of a human action (e.g., sudden movement, picking the dog up). Human negative behavioral responsivity clustered around frustration when the dog employed repeated avoidance behaviors.

To the best of the authors’ knowledge, this is the first attempt to deploy similar devices collecting physiological signals from interacting humans and animals continuously and simultaneously during a CAI session. As such, our study represents a novel attempt to use multiple, synchronized modalities when analyzing human and canine dyads as they interact. Beyond alternate physiological evaluation approaches, replication with larger sample sizes, expanding to other human–animal interactions, and employing more longitudinal data collections, our future work will include generating a stable, open-access data analysis pipeline and repository for CAIs. Such a tool would be beneficial to other similarly disposed researchers and is crucial to standardizing psychophysiological approaches across the animal–computer interaction field. The authors are very interested in appropriately de-identifying this dataset and releasing it for further scientific analysis, extension, and replication. Of special interest are further efforts to integrate appropriate wearable and non-contact systems into a modular toolkit for widespread research use. Consistent equipment, methodology, and data analysis/reporting within human–animal interaction psychophysiology could significantly advance the field’s understanding of interspecies bonding. Lastly, inclusion of contextual and ambient environmental data via the complex analysis of dog smart collar data and the potential inclusion of eye-tracking systems are other promising avenues of future investigation. The former, especially, would complete the multimodal picture of CAIs, which currently only accounts for human and canine subject-centered measurement modalities (i.e., psychological, behavioral, and physiological) and the subsequent points of interaction, while ignoring the broader ambient environmental context. Though some outcomes were counterintuitive and some limitations have been noted, the work herein still represents a uniquely significant step forward on a promising path towards the CAI field’s better understanding of how interactions improve interspecies well-being across time.

## Figures and Tables

**Table 1:** Ergonomics human results table. *: SySy = custom wrist + custom chest + Empatica E4; **: CoSy = Qardio arm + MUSE headband + Biostrap + GoBe2 + Polar H10.

Human Ergonomics		SySy *	n = 21	CoSy **	n = 2	Custom Wrist	n = 4	Custom Chest	n = 4	Empatica E4	n = 5	Zephyr Belt	n = 3	Oura Ring	n = 4
	**Question**	**Mean**	**SD**	**Mean**	**SD**	**Mean**	**SD**	**Mean**	**SD**	**Mean**	**SD**	**Mean**	**SD**	**Mean**	**SD**
General Questions	1	4.67	0.58	5.00	0.00	5.00	0.00	3.75	1.26	5.00	0.00	4.67	0.58	5.00	0.00
	2	4.57	0.75	4.50	0.71	4.75	0.50	4.50	0.58	4.80	0.45	3.00	1.73	3.00	1.41
	3	4.81	0.40	4.50	0.71	4.25	1.50	3.75	1.26	4.40	1.34	3.00	1.73	2.50	1.00
	4	4.76	0.44	4.00	1.41	4.25	1.50	2.25	1.26	4.00	1.22	3.33	1.53	3.25	2.06
	5	4.14	1.06	4.50	0.71	4.50	0.58	3.50	1.00	4.60	0.55	4.33	0.58	2.25	1.50
	6	4.95	0.22	4.50	0.71	4.75	0.50	4.50	0.58	4.80	0.45	4.67	0.58	4.25	0.96
	7	4.71	0.56	4.50	0.71	4.50	1.00	4.00	0.82	4.20	0.84	3.67	1.15	3.50	1.29
	8	4.95	0.22	4.50	0.71	4.50	1.00	4.50	1.00	4.60	0.89	4.33	1.15	4.50	1.00
	9	4.86	0.36	4.50	0.71	4.50	1.00	4.25	0.96	4.60	0.89	3.33	0.58	4.50	1.00
	10	4.76	0.54	4.50	0.71	5.00	0.00	4.50	0.58	5.00	0.00	5.00	0.00	3.00	2.31
Upper Limb Devices	11	4.57	0.98	5.00	0.00	4.50	0.58	–	–	4.60	0.55	–	–	4.50	0.58
	12	4.71	0.90	4.00	1.41	4.75	0.50	–	–	4.60	0.55	–	–	4.75	0.50
	13	4.81	0.51	3.50	2.12	4.75	0.50	–	–	4.40	0.55	–	–	2.50	1.73
Lower Limb Devices	14	5.00	0.00	–	–	–	–	–	–	–	–	–	–	–	–
	15	5.00	0.00	–	–	–	–	–	–	–	–	–	–	–	–
	16	5.00	0.00	–	–	–	–	–	–	–	–	–	–	–	–
Torso Devices	17	4.35	0.88	5.00	–	–	–	3.00	1.15	–	–	4.67	0.58	–	–
	18	4.95	0.22	5.00	–	–	–	5.00	0.00	–	–	5.00	0.00	–	–
Head and Neck devices	19	–	–	4.00	–	–	–	–	–	–	–	–	–	–	–
	20	–	–	5.00	–	–	–	–	–	–	–	–	–	–	–
Interaction Questions	21	4.81	0.40	5.00	0.00	5.00	0.00	4.75	0.50	5.00	0.00	5.00	0.00	4.00	0.82
	22	4.86	0.65	5.00	0.00	5.00	0.00	5.00	0.00	5.00	0.00	5.00	0.00	4.50	1.00
	23	4.95	0.22	5.00	0.00	5.00	0.00	5.00	0.00	5.00	0.00	5.00	0.00	4.50	1.00
	24	5.00	0.00	5.00	0.00	5.00	0.00	5.00	0.00	5.00	0.00	5.00	0.00	4.50	1.00
Overall Percentage Score		95.64		87.73		94.12		84.06		93.65		86.25		76.47	

**Table 2:** Ergonomics canine results table. *: SySy = custom harness + custom smart collar.

Canine Ergonomics		SySy *	n = 21	Custom Harness	n = 5	Custom Smart Collar	n = 4	MeasureON!	n = 2
	**Question**	**Mean**	**SD**	**Mean**	**SD**	**Mean**	**SD**	**Mean**	**SD**
General Questions	1	4.60	0.60	4.20	0.84	5.00	0.00	4.00	0.00
	2	4.50	1.15	4.20	0.84	5.00	0.00	3.00	1.41
	3	4.60	0.60	3.40	1.52	5.00	0.00	2.50	2.12
	4	4.60	0.75	3.40	1.34	4.50	0.58	3.00	2.83
	5	3.80	1.11	4.00	1.00	5.00	0.00	2.00	1.41
	6	4.85	0.37	4.20	0.84	5.00	0.00	4.00	1.41
	7	3.75	1.16	3.60	1.34	5.00	0.00	3.00	0.00
	8	4.65	0.81	3.60	1.52	5.00	0.00	3.50	2.12
	9	4.45	0.60	3.80	1.30	5.00	0.00	3.50	2.12
	10	4.30	1.03	4.60	0.89	5.00	0.00	4.50	0.71
	11	4.70	0.73	4.40	0.55	5.00	0.00	3.50	0.71
Torso Devices	12	4.70	0.73	4.40	0.89	–	–	3.50	0.71
	13	4.75	0.72	4.20	0.84	–	–	4.00	0.00
Head & Neck Devices	14	4.83	0.51	–	–	5.00	0.00	–	–
	15	4.89	0.47	–	–	5.00	0.00	–	–
Interaction Questions	16	4.45	0.83	3.80	0.84	5.00	0.00	3.00	1.41
	17	3.40	1.05	2.40	1.52	4.75	0.50	2.50	0.71
	18	4.80	0.70	3.80	0.84	5.00	0.00	3.50	0.71
	19	4.95	0.22	4.00	1.22	5.00	0.00	5.00	0.00
Overall Percentage Score		90.08		77.65		99.12		68.24	

**Table 3:** Neutral session to interaction session summary statistics and comparisons for the SAM and PANAS surveys. SAM-V = valence; SAM-A = arousal; PANAS-PA = positive affect; PANAS-NA = negative affect; sd = standard deviation; INT1 = interaction session 1; INT2 = interaction session 2; NEU = neutral session; BASE = baseline session; POST = postline session; ALL NEU = all neutral sessions; ALL INT = all interaction sessions.

Subsession	Statistic	SAM-V	SAM-A	PANAS-PA	PANAS-NA
BASE	mean	2.32	3.64	21.05	11.73
	sd	0.57	1.09	5.14	2.75
INT1	mean	2.18	3.09	21.82	11.18
	sd	0.91	0.97	6.45	2.28
NEU	mean	2.33	4.00	18.22	10.67
	sd	0.59	0.97	4.60	1.37
INT2	mean	2.38	3.15	19.23	11.15
	sd	0.51	0.90	6.08	1.46
POST	mean	2.33	3.67	18.83	10.67
	sd	0.52	1.03	5.85	0.82
All NEU	mean	2.33	3.78	19.65	11.17
	sd	0.56	1.03	5.1	2.14
All INT	mean	2.26	3.11	20.86	11.17
	sd	0.78	0.93	6.35	1.99
ALL NEU vs. ALL INT	Wilcoxon *p* value	0.043	0.0002	0.0003	0.0755

**Table 4:** Average behavioral coded state across all interaction sessions and subjects. h- = for human subjects; c- = for canine subjects; INT1 = interaction session 1; INT2 = interaction session 2; pos = positive code; neu = neutral code; neg = negative code.

	pos %	neu %	neg %
**h-INT1**	16.03	83.26	0.25
**c-INT1**	23.22	63.84	0.92
**h-INT2**	11.67	87.60	0.23
**c-INT2**	12.65	64.38	1.23

**Table 5:** Physiological data summary table. NEU = neutral session; INT = interaction session; S+1m = session start plus 1 min; M = middle of session; E-1m = session end minus 1 min; CSens = custom-designed sensor on human; wCSens = custom sensor on wrist; cCSens = custom sensor on chest; HAR = harness (on dog chest); E4 = Empatica E4 (on human wrist); HR = heart rate; SDNN = standard deviation of NN intervals; RMSSD = root mean square of successive differences between heartbeats; SKIN Temp = skin temperature; EDA Mean = average electrodermal activity by epoch; EDA Max = maximum electrodermal activity by epoch; EDA Peak Ct = number of peaks in epoch of electrodermal activity; IMA = integral modulus of acceleration.

s_s type	s_s time	CSens_HR	CSens_ SDNN	CSens_ RMSSD	HAR_HR	HAR_ SDNN	HAR_ RMSSD	E4_Temp	E4_EDA Mean	E4_EDA Max	E4_EDA Peak Ct	wCSens_ IMA	cCSens_ IMA	HAR_IMA	E4_IMA
NEU1	S+1m	80.2	5.54	17.43	98.5	2.15	2.90	21.9	0.001	0.002	0.714	1.350	1.582	1.589	1.512
	M	88.6	7.65	11.92	81.4	4.43	0.36	23.6	0.934	1.009	0.719	1.350	1.582	1.589	1.512
	E−1m	74.9	1.88	2.07	96.1	4.98	9.58	26.1	1.470	1.566	0.702	1.350	1.582	1.589	1.512
INT1	S+1m	74.3	2.68	3.15	96.1	14.26	24.76	29.4	2.086	2.203	.0750	1.369	1.584	1.589	1.510
	M	73.1	2.98	4.02	90.1	7.50	11.76	32.3	1.921	2.070	0.743	1.369	1.584	1.589	1.510
	E−1m	76.3	5.66	7.06	95.2	12.47	18.62	32.5	2.440	2.677	0.619	1.370	1.584	1.589	1.511
NEU2	S+1m	70.4	5.02	5.04	103.0	8.27	11.67	32.7	2.274	2.457	1.012	1.386	1.546	1.590	1.490
	M	71.5	5.12	5.59	94.6	10.39	14.24	33.0	2.143	2.237	0.983	1.386	1.546	1.590	1.489
	E−1m	74.2	5.60	7.30	90.3	11.03	17.46	33.2	1.669	1.728	0.726	1.386	1.546	1.590	1.489
INT2	S+1m	71.3	5.38	4.85	86.5	13.88	20.99	33.4	1.990	2.048	0.900	1.317	1.569	1.619	1.478
	M	72.4	5.85	6.42	90.0	12.85	19.02	33.4	2.385	2.537	1.017	1.317	1.574	1.619	1.478
	E−1m	64.8	7.34	6.80	85.6	15.47	27.62	33.5	2.059	2.236	1.250	1.317	1.566	1.619	1.478
NEU3	S+1m	73.1	10.49	13.86	89.7	14.21	21.88	33.2	1.288	1.387	0.867	1.359	1.488	1.622	1.452
	M	79.3	27.83	42.37	97.2	15.97	23.46	33.1	1.593	1.707	1.003	1.359	1.488	1.622	1.452
	E−1m	83.2	27.80	34.22	81.9	14.07	22.91	32.9	2.762	2.947	0.983	1.359	1.488	1.622	1.452
Wilcoxon	*p*-value	0.37	0.79	0.97	0.04	0.83	0.71	0.00001	0.01	0.01	0.75	0.37	0.43	0.21	0.11

**Table 6:** Behavioral coding subset table [72]. INT = interaction session; H = human subject; C = canine subject; Physio = physiological measures; HR = heart rate; SDNN = standard deviation of NN intervals; RMSSD = root mean square of successive differences between heartbeats; IMA = integral modulus of acceleration; Pcorr = Pearson’s correlation; DTW = dynamic time warping; Becode = behavior coding; h = human; c = canine; pos = positive code; neg = negative code; neu = neutral code; PANAS-PA = positive affect; PANAS-NA = negative affect; SAM-V = valence; SAM-A = arousal.

INT1		H1–C1						H2–C1						H3–C1				
	**Physio**	**HR**	**SDNN**	**RMSSD**	**IMA**		**Physio**	**HR**	**SDNN**	**RMSSD**	**IMA**		**Physio**	**HR**	**SDNN**	**RMSSD**	**IMA**	
	Pcorr	−0.564	−0.291	−0.282	−0.032		Pcorr	−0.289	0.359	0.432	−0.079		Pcorr	−0.032	0.214	0.127	−0.007	
	DTW	2202.1	23.4	50.9	297.2		DTW	16,461.6	81.3	109.6	1.0		DTW	21,456.5	31.1	58.8	65.9	
	Becode	% same	pos %	neu %	neg %		Becode	% same	pos %	neu %	neg %		Becode	% same	pos %	neu %	neg %	
	h	21.38	30.88	69.12	0		h	56.10	18.67	80.00	1.33		h	86.84	1.41	98.59	0	
	c	--	51.47	48.53	0		c	--	14.67	73.33	1.33		c	--	0	94.37	5.63	
	Survey	MDORS	SAM-V	SAM-A	PANAS-PA	PANAS-NA	Survey	MDORS	SAM-V	SAM-A	PANAS-PA	PANAS-NA	Survey	MDORS	SAM-V	SAM-A	PANAS-PA	PANAS-NA
	Pre	87	3	2	22	19	Pre	98	2	3	15	10	Pre	131	2	5	23	12
	Post	--	4	2	30	10	Post	--	3	4	12	10	Post	--	2	4	16	10
**INT2**		**H1–C1**						**H2–C1**						**H3–C1**				
	**Physio**	**HR**	**SDNN**	**RMSSD**	**IMA**		**Physio**	**HR**	**SDNN**	**RMSSD**	**IMA**		**Physio**	**HR**	**SDNN**	**RMSSD**	**IMA**	
	Pcorr	−0.236	−0.023	−0.001	0.007		Pcorr	0.169	0.198	0.197	0.061		Pcorr	0.232	−0.122	0.033	−0.002	
	DTW	44,020.7	634.7	829.7	64.3		DTW	34,503.5	73.2	136.9	276.6		DTW	56,624.3	56.9	150.4	640.2	
	Becode	% same	pos %	neu %	neg %		Becode	% same	pos %	neu %	neg %		Becode	% same	pos %	neu %	neg %	
	h	37.11	86.57	13.43	0		h	71.05	10.67	89.33	0		h	52.20	0	100	0	
	c	--	97.01	1.49	0		c	--	21.33	74.67	4		c	--	1.16	96.51	2.33	
	Survey	MDORS	SAM-V	SAM-A	PANAS-PA	PANAS-NA	Survey	MDORS	SAM-V	SAM-A	PANAS-PA	PANAS-NA	Survey	MDORS	SAM-V	SAM-A	PANAS-PA	PANAS-NA
	Pre	87	3	3	24	10	Pre	98	2	3	12	10	Pre	131	2	4	21	10
	Post	--	1	2	32	10	Post	--	2	3	13	10	Post	--	3	5	19	10

## Data Availability

The datasets presented in this article are not readily available, due to the data being part of an ongoing study and to technical and time limitations. Requests to access the datasets should be directed to the corresponding author.

## Supplementary Materials

The following supporting information can be downloaded at: https://www.mdpi.com/article/10.3390/ani14243628/s1.
