# Supplementary material for: Exploring the Dynamics of Canine-Assisted Interactions: A Wearable Approach to Understanding Interspecies Well-Being
Source: Animals (Basel). 2024 Dec 16;14(24):3628. doi: 10.3390/ani14243628 (PMC11672835; doi:10.3390/ani14243628)
Supplement: Supplementary file 1 [file animals-14-03628-s001.zip › animals-3279623-supplementary.pdf]

Exploring the Dynamics of Canine-Assisted Interactions: A Wearable Approach to Understanding Interspecies Well-Being

Supplementary Materials

Table of Contents

Section S1 -- Stakeholder Survey
- Table S1
- Table S2
- Figure S1

Section S2 -- Device Characterization
- Figure S2
- Table S3
- Table S4
- Table S5
- Table S6

Section S3 -- Ergonomics
- Figure S3
- Figure S4
- Figure S5
- Figure S6
- Figure S7
- Figure S8

Section S1 -- Stakeholder Survey

Table S1

Table S1: Likert scale responses of CAI Stakeholders to Section #1 General Questions.

S1. Questions	Mean	SD
1. How satisfied are you with Canine Assisted Interactions generally?	3.7	0.95
2. What is your satisfaction with current methods of measuring Psychophysiological (PP) states in CAI? (*e.g.* psychological surveys, behavior coding, biochemical analytes, *etc.*)	2.6	1.07
3. What is your level of satisfaction with the outcomes of the interactions for humans? (*i.e.* is the patient happier, less fatigued, *etc.*)	3.7	0.82
4. What is your satisfaction with outcomes of the interactions for canines?	2.7	0.82
5. How familiar are you with the current landscape of technologies for quantifying human-dog interactions?	3.1	1.60
6. How much do you think ambient environment affects CAI interactions? (*i.e.* room temperature, relative humidity, *etc.*)	3.8	1.14
7. How much do you think social environment affects CAI interactions? (*i.e.* number of people in the room, other animals present, *etc.*)	4.7	0.48
8. How optimistic are you for the future of CAI's generally?	4.3	0.67

Table S2

Table S2: Short response questions for Sections #2-4 of the stakeholder survey.

S2. psychophysiological State Identification Questions
9. When observing a dog what do you think a dog's positive psychophysiological state (i.e. happy, excited, increasing QoL, etc.) would look like? What would a negative

psychophysiological state (i.e. fearful, depressed, decreasing QoL, etc.) look like?

10. Do you have any experience in measuring well-being/psychophysiological state in dogs? If yes, what methodologies, devices or other measures did you use? Did these tools adequately serve your purposes? [Please be specific (i.e. owner surveys, wearables, implantables, non-contact systems, behavior coding, biomarker assays, etc.)]

11. Considering your response to the previous question, what tools do you generally feel you need or would be nice to have in the CAI field?

12. Continuing on from Questions #10 & #11, in what forms should the data from these desired tools come? What decisions would you seek to make with this data? What workarounds do you currently use to achieve your goals in lieu of this desired data?

S3. CAI Context & Design Questions

13. In your most typical CAI use cases, who is present (e.g. therapy animals, handlers, medical staff, other patients/participants, etc.)? What is the CAI interaction room & context (e.g. hospital waiting room for patients before an appointment)? How long (in minutes) is the human-dog intervention typically?

14. What are your primary ergonomic considerations for therapy dogs wearing electronics or other research devices during CAIs? For humans?

15. In your opinion, what are the 5 best, repeatable CAI activities to induce a positive psychophysiological state in the therapy dog? In the human participant?

Table S2: Short response questions for Sections #2-4 of the stakeholder survey (continued).

**S3. CAI Context & Design Questions (continued)**<br>16. If you were to design a human-dog interaction with the sole goal of maximizing mutual wellbeing, what would you suggest be done by researchers or subjects before, during, and after the interaction? (After listing your items, please rank them by importance.)
**S4. SySy Evaluation Questions***<br>17. What are your thoughts on how to determine "causal attribution" generally in CAIs? Specifically by using the SySy system?<br>18. What are your thoughts on how to approach "individualization" generally in CAIs? Specifically by using the SySy system?<br>19. What are your thoughts on how to approach "benefit enhancement" generally in CAIs? Specifically by using the SySy system?<br>20. What joint human/canine activities do you facilitate? Are you open to joint experiments with our research lab? How could/would we incorporate SySy into your activities to provide meaningful information?<br>21. How would you recommend we go about identifying and recruiting the desired participant populations (i.e. therapy dyads, pet owners, etc.) to evaluate the SySy and/or provide your desired CAI data?<br>22. Considering our goals, do you have any final thoughts or suggestions? (e.g. system improvements, suggested experiments, suggested data analysis, additional subject populations worth considering, etc.)?

The 'SySy Evaluation Questions' were preceded by the context primer in **Figure S1.3**

Figure S1

SySy Evaluation Questions

*** Please Read Before Continuing***

The iBionics lab led by Dr. Alper Bozkurt is developing a Synchronized System (SySy) of electronic devices, psychological instruments, and behavioral methods to advance the state of the practice for CAI psychophysiology.

This work aims to identify psychophysiological changes and data features observed by wearable and non-contact devices during CAIs with therapy dyads as research models of healthy human subject interaction with one's pet. More specifically, we aim to monitor multiple physiological sensing modalities (e.g., heart rate (HR), activity level, skin temperature (ST), etc.) using several commercial devices as well as three prototypes developed by NC State researchers. Surveys and subject behavioral coding are integrated into this pipeline to help us evaluate the usefulness of our Synchronized System in CAIs.

Ultimately, we hope the products of this work will significantly advance the CAI field by permitting causal attribution, individualization, and benefit enhancement.
- Causal attribution is the act of connecting interaction inputs (e.g. human offers a treat, dog jointly plays with a toy, the room is a pleasant temperature, etc.) to interaction outcomes (e.g. dog is excited, human is happy, improvement in patient vital signs, etc.)
- In the CAI context, individualization is tailoring the context and activities of a canine-assisted interaction to the receiving human participant (e.g. a quiet, calm interaction for a hospitalized individual vs. an active, play session for a child in school, etc.)
- Benefit enhancement refers to the process of directly trying to increase or maximize positive PP state and/or other beneficial outcomes for either or both parties in a human-canine interaction.

With these aims, methods and definitions in mind, please answer the following questions.

Figure S1. The contextualization prompt that preceded the 'SySy Evaluation Questions.

Section S2 -- Device Characterization

Figure S2

Figure S2: Wearable research system devices, representation, and data streams.

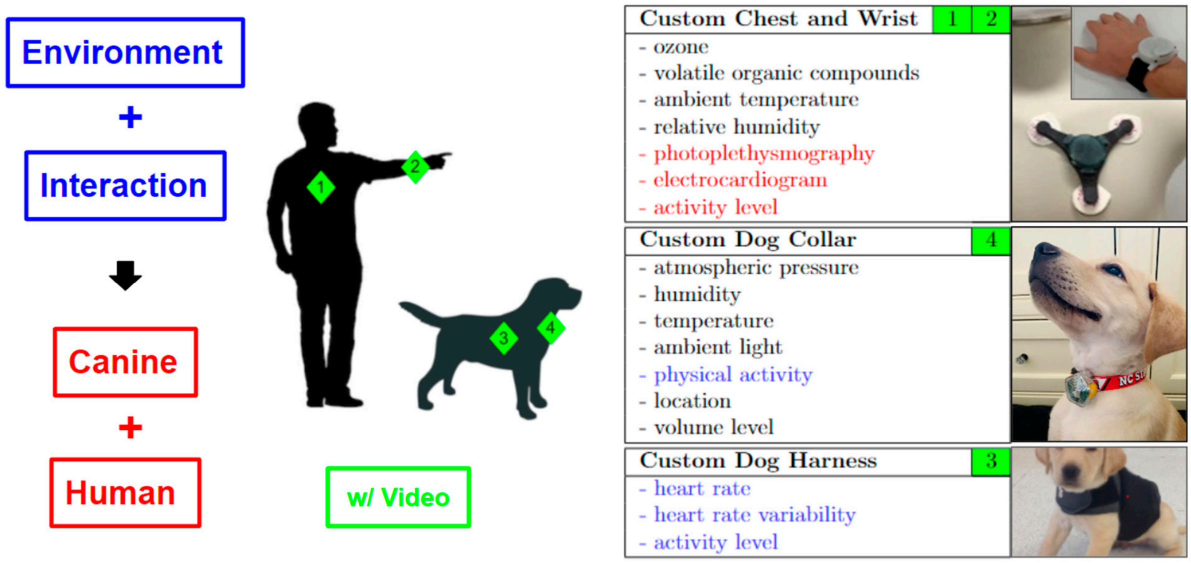

The chart on the left depicts the involved CAI data streams. The dog human silhouette depicts the location of each system device. The representative pictures and paired lists further depict each system's location and sensors.

Table S3

Table S3: Sample heart rate test results for GEB Harness, HET chest patch and Empatica E4.

	HET Chest patch	GEB Harness	Empatica E4
Device HR	85.25	87.62	80.56
Vernier HR	84.94	88.18	85.88
% abs error	0.36%	0.64%	6.19%

Table S4

Table S4: Summary of average wearable system device connectivity characteristics.

Device	Distance b4 disconnect	Distance to reconnect

E4	167 ft	147 ft
GEB Harness	150* ft	--
GEB smart collar	72 ft	68 ft
HET Chest	63 ft	58 ft
HET Wrist	60 ft	57 ft
No GEB Harness disconnect in 150 ft.		

Table S5

Table S5: Wearable system devices Received Signal Strength Indicator (RSSI) for BLE and WiFi at various distances.

	RSSI Range (in dB/m)					
	HET Chest	HET Wrist	GEB Smart Collar	GEB Harness	Empatica E4	iOS Aggregator
0 feet	-42 to -54	-52 to -57	-45 to -52	-48 to -52	-39 to -47	-28 to -49
20 feet	-75 to -95	-85 to -95	-92 to -100	-56 to -60	-78 to -80	-59 to -66
40 feet	-85 to -95	-88 to -92	-90 to -100	-71 to -78	-80 to -89	-59 to -80
60 feet	-85 to -95	-88 to -100	--	--	--	--
80 feet	-90 to -100	-95 to -105	--	--	--	--

Table S6

Table S6: Summary of battery characteristics during charge and discharge of wearable system devices.

	**Charge**	**Discharge**
GEB Smart Collar	3 hrs 23 mins	12 hrs 52 mins
GEB Harness	--	--
HET Chest	1 hr 9 mins	13 hrs 27 mins
HET Wrist	2 hrs 11 mins	11 hrs 36 mins
Empatica E4	1hr 29 mins	27 hrs 44 mins

Section S3 -- Ergonomics

Figure S3

Human Wearer Activity

1. Please put on the device/s according to the appropriate provided instructions.
2. Please complete the following activities.
    (a) 5 jumping jacks, 5 torso twists, 5 stand and sits, walk 10 feet away and return to start, 5 wrist and ankle rotations
    (b) interact with a stuffed/dog for 2 minutes

Canine Wearer Activity

1. Please think, for 2 minutes, about the typical signs this dog shows to express discomfort or pain.
2. Please put the device/s on the dog according to the appropriate provided instructions.
3. Please monitor the dog for 15 minutes paying special attention to the following activities.
    (a) walking, running, sitting, lying down, play activities, self-grooming, other grooming (e.g. brushing,) vocalization, scratching or discomfort behaviors, etc.

Figure S3: Ergonomics device test protocol.

Figure S4

A. General Questions
1. The device is intuitive to put on/take off.
2. The device is intuitive to operate.
3. The device fits me well.
4. The device stays in place at all times.
5. The device is comfortable to wear.
6. The device is/feels safe to wear.
7. The device does NOT limit my mobility.
8. The device does NOT limit my energy.
9. The device does NOT generate any negative emotions.
10. The device does NOT cause me any physical pain.
(Add'l comments for this sub-section)

B. Upper Limb devices
11. The device does NOT catch or knock against other objects/clothing.
12. The device does NOT add noticeable weight to my upper limb/s.
13. Wearing the device, my fingers, hand, wrist, elbow, upper arm, lower arm, and shoulder have normal range of motion.

C. Lower Limb devices
14. The device does NOT catch or knock against other objects/clothing.
15. The device does NOT add noticeable weight to my lower limb/s.
16. Wearing the device, my toes, feet, ankles, knees, legs, and waist have normal range of motion.

D. Torso devices
17. The device does NOT catch or knock against other objects/clothing.
18. The device does NOT add noticeable weight to my torso.

E. Head & neck devices
19. The device does NOT add noticeable weight to my head.
20. Wearing the device, my head and neck have normal range of motion.

F. Interaction Questions
21. The device allows me to play with the dog normally.
22. The device allows me to groom the dog normally.
23. The device allows me to otherwise interact with the dog normally.
24. The device allows the dog to respond normally to the interaction.

Figure S4: Ergonomics Human Survey Questions.

Figure S5

A. General Questions
1. The device is intuitive to put on/take off.
2. The device is intuitive to operate.
3. The device fits the dog well.
4. The device stays in place at all times.
5. In my opinion, the device is comfortable for the dog to wear.
6. In my opinion, the device is/feels safe for the dog to wear.
7. The device does NOT alter the dog's normal behavior.
8. The device does NOT disturb the dog's normal rest/ing positions.
9. The device does NOT limit the dog's normal mobility.
10. The device does NOT limit the dog's energy.
11. The device does NOT cause the dog any physical pain.
(Add'l comments for this sub-section)

B. Torso Devices
12. During wear, the device does NOT catch or knock against other objects in the dog's environment.
13. The device does NOT add noticeable weight to the dog's torso.

C. Head & neck devices
14. The device does NOT add noticeable weight to the dog's head.
15. Wearing the device, the dog's head and neck have normal range of motion.

D. Interaction Questions
16. The device allows me to play with the dog normally.
17. The device allows me to groom the dog normally.
18. The device allows me to otherwise interact with the dog normally.
19. The device allows the dog to respond normally to the interaction.

Figure S5: Ergonomics Canine Survey Questions.

Figure S6

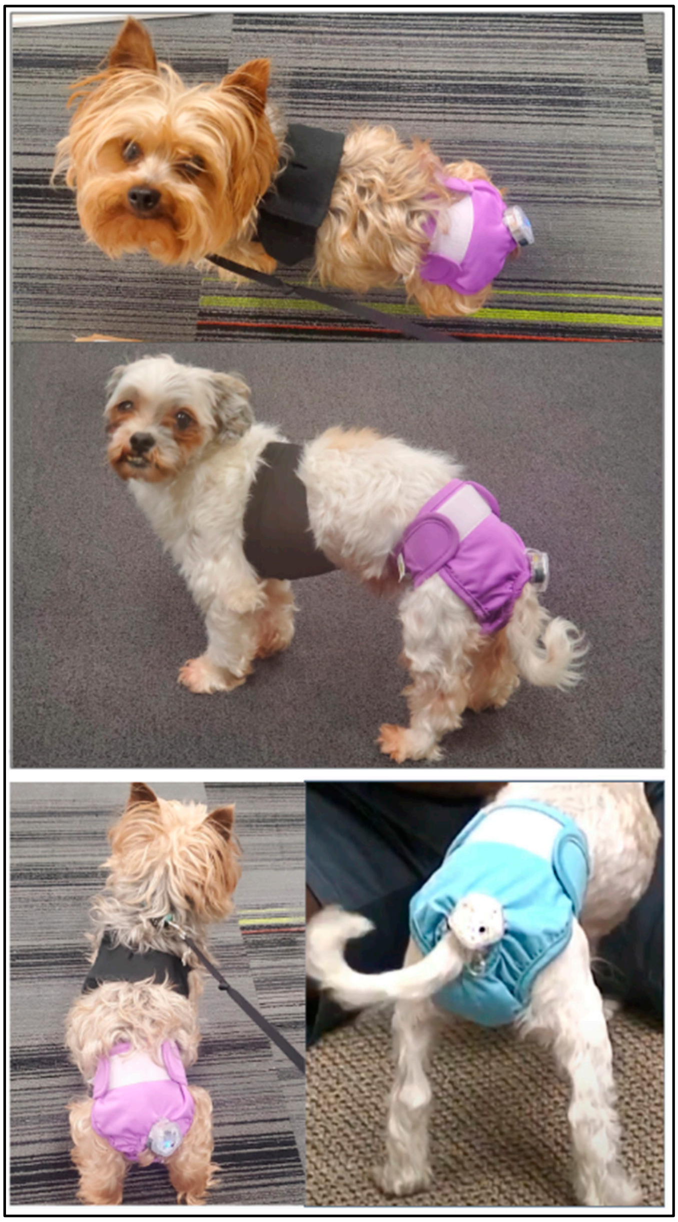

Figure S6: Picture of dog-diaper mounted tail wag sensor.

Figure S7

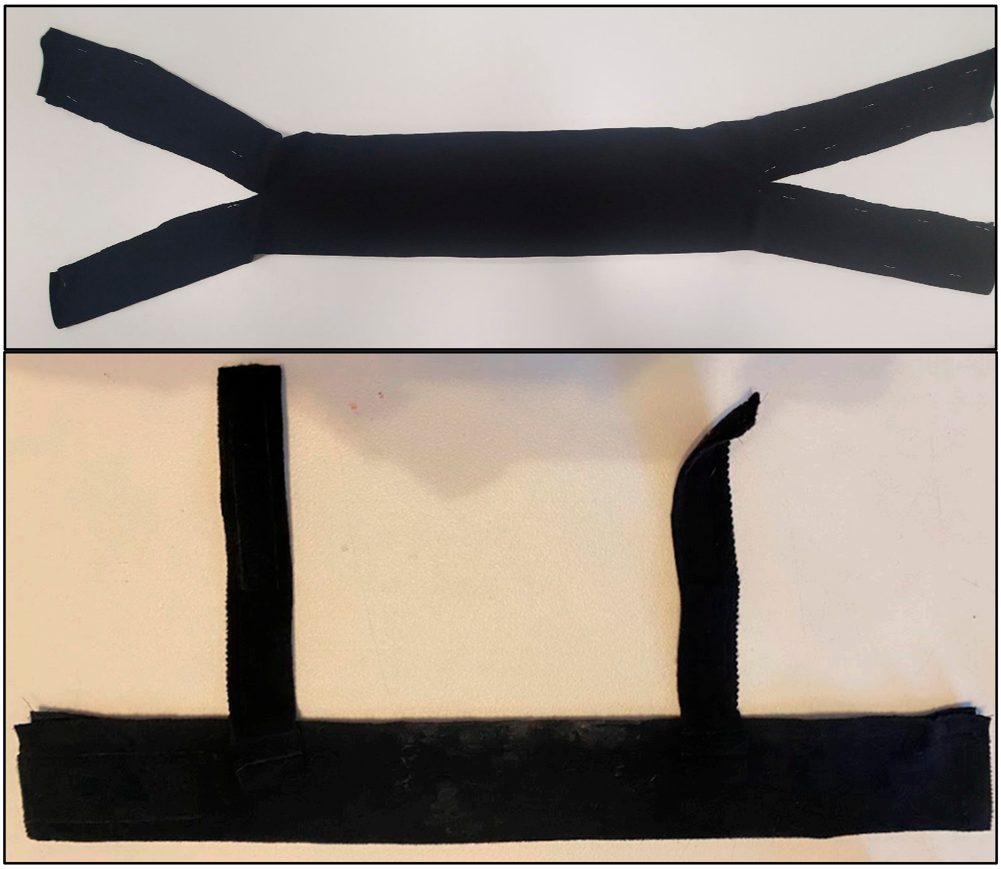

Figure S7: Updated dog harness

Figure S8

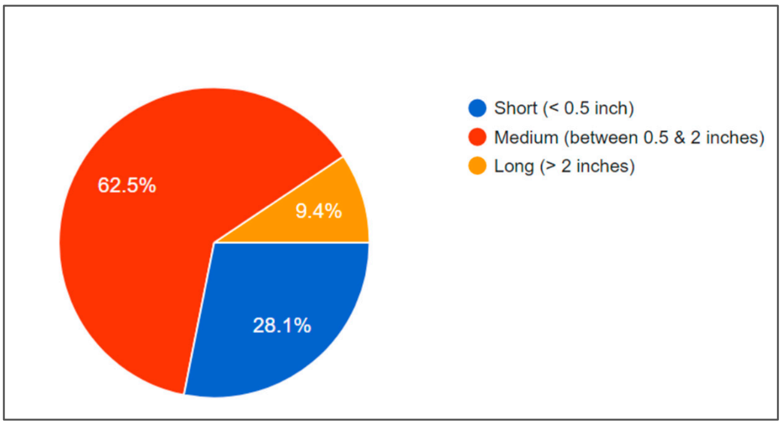

Figure S8: Dog Fur length Chart.
